# SIRT6 in Senescence and Aging-Related Cardiovascular Diseases

**DOI:** 10.3389/fcell.2021.641315

**Published:** 2021-03-29

**Authors:** Xiaokang Li, Lin Liu, Tian Li, Manling Liu, Yishi Wang, Heng Ma, Nan Mu, Haiyan Wang

**Affiliations:** ^1^Department of Cardiology, China Tangdu Hospital, Fourth Military Medical University, Xi’an, China; ^2^Department of Dermatology, Tangdu Hospital, Fourth Military Medical University, Xi’an, China; ^3^Department of Physiology and Pathophysiology, School of Basic Medicine, Fourth Military Medical University, Xi’an, China

**Keywords:** SIRT6, senescence, cardiovascular diseases, autophagy, oxidative stress

## Abstract

SIRT6 belongs to the nicotinamide adenine dinucleotide (NAD^+^)-dependent deacetylases and has established diverse roles in aging, metabolism and disease. Its function is similar to the *Silent Information Regulator 2 (SIR2)*, which prolongs lifespan and regulates genomic stability, telomere integrity, transcription, and DNA repair. It has been demonstrated that increasing the sirtuin level through genetic manipulation extends the lifespan of yeast, nematodes and flies. Deficiency of SIRT6 induces chronic inflammation, autophagy disorder and telomere instability. Also, these cellular processes can lead to the occurrence and progression of cardiovascular diseases (CVDs), such as atherosclerosis, hypertrophic cardiomyopathy and heart failure. Herein, we discuss the implications of SIRT6 regulates multiple cellular processes in cell senescence and aging-related CVDs, and we summarize clinical application of SIRT6 agonists and possible therapeutic interventions in aging-related CVDs.

## Introduction

Population ageing is a global phenomenon. Virtually every country in the world is experiencing growth in the size and proportion of older persons in their population (United Nations, 2019). With the growing of aged population, the incidence of aging related cardiovascular diseases (CVDs) is increasing. According to a report from the American Heart Association, CVDs (comprising coronary heart disease, heart failure, stroke, and hypertension) currently claims more lives each year than cancer and chronic lung disease combined, and the prevalence of CVD in adults ≥ 20 years of age is 48.0% overall and increases with age in both males and females (Virani et al., 2020). Therefore, aging is an independent risk factor associated with the progressive degeneration of the heart, making them more vulnerable to stressors and contributing to increased morbidity and mortality (Chiao and Rabinovitch, 2015).

**FIGURE 1:**
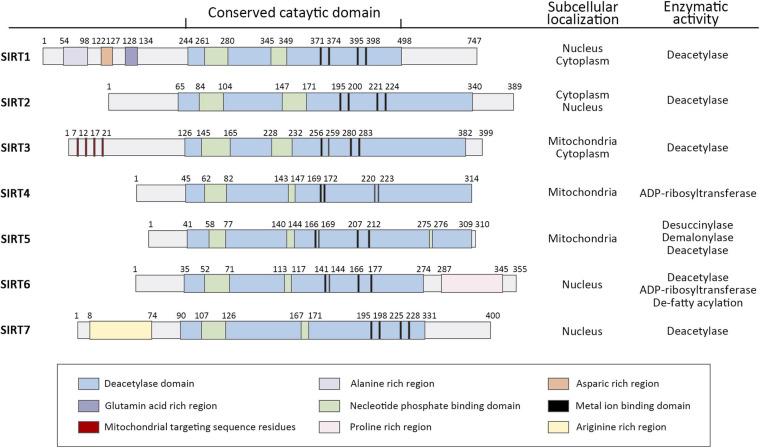
Domain architecture, subcellular localization, and enzymatic activity of human sirtuin family of Class III NAD^+^-dependent histone deacetylases. Schematics represent the domain structure of human sirtuins. Amino acid positions are noted above each schematic. The domains are represented in different colors. Adapted from UniProt Universal Protein Resource Database.

It has been probably 20 years since the Silent Information Regulator 2 (SIR2) gene was found to extend the lifespan of yeast (Kaeberlein et al., 1999). From that time on, it sparked efforts in many institutions to realize more SIR2-like genes, known as sirtuins, and elucidate their potential to delay the onset of age-related diseases. Sirtuins are a family of histone deacetylases (HDACs) that catalyze deacetylation of both histone and non-histone lysine residues. Their requirement for nicotinamide adenine dinucleotide distinguishes sirtuins from other HDAC classes and defines them as class III HDACs (Winnik et al., 2015). Mammals contain seven sirtuins (Figure 1), SIRT1-7, which are categorized by their different subcellular localization, unique binding substrates and diverse enzymatic activities (Haigis and Sinclair, 2010). These members share a conserved catalytic domain spanning 250 amino acids. The catalytic core comprises an NAD^+^-binding domain and four structural zinc-binding domains. Catalysis occurs in a hydrophobic cleft or pocket situated between these two kinds of domains and the hydrophobic cleft or pocket often provides binding sites for modulators. Additionally, sirtuins contain diverse N and C terminal extensions that can direct cellular localization and protein-protein interactions.

The member SIRT6 is widely expressed in all mammalian organs and regulates multiple senescence associated biological processes, including oxidative stress, glucose and fat homeostasis (Mostoslavsky et al., 2006; Zhong et al., 2010; Tao et al., 2013a), inflammatory responses, autophagy, genome integrity, and telomeres homeostasis (Papamichos-Chronakis and Peterson, 2013; Kugel and Mostoslavsky, 2014; Miller and Sadeh, 2014; Lasry and Ben-Neriah, 2015; Tasselli et al., 2017; Abdellatif et al., 2018; De Meyer et al., 2018). Therefore, SIRT6 is involved in many kinds of aging related disease such as neurodegenerative disease, cancer, CVDs. For instance, recent study demonstrated that SIRT6−deficient cynomolgus monkeys exhibit developmental retardation (Zhang W. et al., 2018). However, in Alzheimer’s disease (AD) patients, SIRT6 plays AD−protective function via maintaining genomic stability and preventing DNA damage in brain (Jung et al., 2016; Kaluski et al., 2017). It not only reveals a pivotal role in brain development, but also shows a close relationship between the aberration of SIRT6 with human neurodegenerative diseases. In the context of cancer, SIRT6 was considered as a double−edged sword due to its dual role of both tumor suppression and promotion, depending on the type of tumors (Desantis et al., 2017). It protects against tumor growth through the functions of controlling DNA damage repair, genomic stability, cellular metabolic homeostasis, and apoptosis, while it also associated with the poor clinical outcomes by its enzyme activity regulating cancer pathways in cancers such as hepatocellular and colon cancers (Sebastian et al., 2012; Vitiello et al., 2017; Khan R. I. et al., 2018). In the cardiovascular system, SIRT6 plays a protective function by improving vascular endothelial dysfunction to some extent, delaying the formation of atherosclerotic plaques and inhibiting cardiac hypertrophy and heart failure (Sundaresan et al., 2012; Liu et al., 2016). In addition, several studies showed that SIRT6 is a principal regulator of glucose metabolism homeostasis (Zhong et al., 2010; Xiong et al., 2016). Targeting it may be a promising strategy for attenuating diabetic cardiomyopathy (DCM) and reducing myocardial vulnerability to ischemia-reperfusion injury in diabetic patients (Yu et al., 2021).

In this review, we chiefly interrogate the role of SIRT6 in cell senescence, the main CVDs involved in cell senescence induced by SIRT6 dysfunction, and possible clinical application of SIRT6 functional regulatory drugs in CVDs.

## SIRT6 and Senescence

**FIGURE 2:**
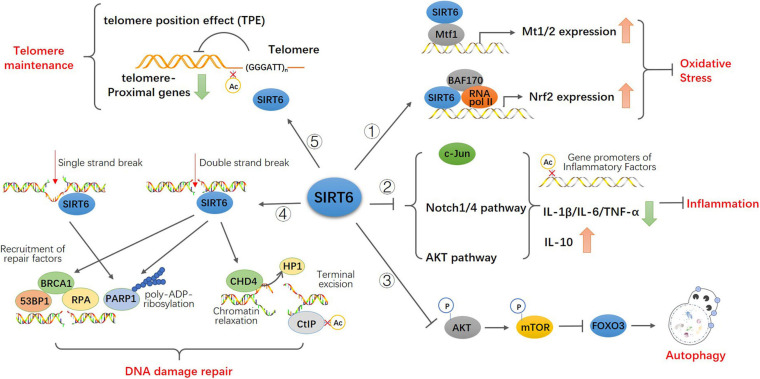
Model for the function of SIRT6 in age-related cellular events. SIRT6 directly binds to Mtf1 to promote the expression of metallothionein Mt1 and Mt2. SIRT6 recruits BAF170 and RNA polymerase II to promote the expression of Nrf2 and downstream genes to participate in antioxidant stress. SIRT6 inhibits the activity of c-JUN, the transcription of Notch1 and Notch4 signals and the phosphorylation of Akt signal via epigenetic regulation. These upregulate the expression of pro-inflammatory cytokines IL-1 β, IL-6, and TNF-α and downregulate anti-inflammatory cytokines IL-10 to attenuates the effect of inflammation. SIRT6 inhibites the activity of Akt-mTOR pathway thus promotes FOXO3-dependent autophagy. Both single and double strand break trigger the recruitment of SIRT6 and activation of PARP1 at the damage sites and promote PARP1 mediated DNA repair. SIRT6 also recruits repair factors 53BP1, BRCA1, and RPA at double-strand breakpoint for damage repair. SIRT6 recruits and interacts with CHD4 to render the relaxation of chromatin required for DNA repair. And CHD4 replaces HP1 in histone H3K9 further promoting homologous recombination. SIRT6 binds to and deacetylate CtIP to promote terminal excision. SIRT6 keeps the low physiological level of H3K9 acetylation and preserves the telomere position effect to maintain normal function of telomere.

Senescence is a multi-factor process involving the regulation of different age-related molecular and cellular events, including oxidative stress and neurodegeneration, glucose and fat homeostasis (Mostoslavsky et al., 2006; Zhong et al., 2010; Tao et al., 2013a), inflammatory responses, autophagy, genome integrity, and telomeres shorten (Papamichos-Chronakis and Peterson, 2013; Miller and Sadeh, 2014; Lasry and Ben-Neriah, 2015; Abdellatif et al., 2018; De Meyer et al., 2018). The role of sirtuins in senescence was discovered in budding yeast, where overexpression of SIR2 increases replicative lifespan. Subsequently, It was reported that elevated sirtuin levels increase lifespan in the nematode *C. elegans* (Tissenbaum and Guarente, 2001) and the fruitfly Drosophila (Rogina and Helfand, 2004), indicating an evolutionarily ancient role of sirtuins in longevity assurance. However, despite recently there have debates about the direct role of SIR2 in aging and lifespan extension, especially in budding yeast and *C. elegans* (Kaeberlein, 2010; Kenyon, 2010), the overwhelming majority of significant results still support a potential role for SIRT6 in regulating mammalian lifespan (Yuan et al., 2009; Burnett et al., 2011; Kanfi et al., 2012). SIRT6 was shown to extend lifespan in mammals, while deficiency of SIRT6 was associated with progeria, an accelerated aging disorder (Liao and Kennedy, 2012, 2014). Studies have confirmed the important roles for SIRT6 in protecting against aging and disease pathologies: SIRT6-deficient mice are small and have severe metabolic defects, and by 2–3 weeks of age, they develop abnormalities that are usually associated with aging (Mostoslavsky et al., 2006). SIRT6-deficient monkeys die hours after birth and exhibit severe prenatal developmental retardation (Zhang W. et al., 2018). However, SIRT6 overexpression led to an increase in lifespan in male mice (Kanfi et al., 2012). Mechanistically, SIRT6, being a deacetylase at the specific site of histone H3K9 H3K56 H3K18 (Michishita et al., 2008, 2009; Tasselli et al., 2016), inhibits the transcription of transcription factors related to senescence, maintains the structure of telomere chromatin, prevents genomic instability after DNA damage, and protects cells from senescence (Tennen and Chua, 2011; Kugel and Mostoslavsky, 2014). Here, we summarized the function of SIRT6 in age-related cellular events (Figure 2).

### SIRT6 and Oxidative Stress

Based on the free radical theory, aging is triggered by a long-term cumulative damage of toxic free radicals and reactive oxygen species (ROS) to sensitive targets with biologically significance. Moreover, early studies revealed that the accumulation of ROS is closely related to the poor prognosis of CVDs (Griendling and FitzGerald, 2003). It is known to all that the maintenance of the heart’s pumping action requires functional and morphological integrity of mitochondria to ensure an uninterrupted energy supply. Meanwhile, mitochondria, as heart’s energy providers, also can generate ROS as a by-product. Recent study indicated that an increase in mitochondrial ROS followed by ultrastructural alterations in the mitochondrial cristae lead to cardiomyocyte damage and, ultimately, cell death (Acin-Perez et al., 2018). Treatment of primary fibroblasts with medium, non-lethal doses of exogenous hydrogen peroxide can activate rapid, senescence-like growth arrest (Chen and Ames, 1994). Analogously, cells grown in the company of high oxygen concentrations exhibit a reduced lifespan and show telomeres shorten (von Zglinicki et al., 1995).

Reports indicate that SIRT6 is highly sensitive to cellular redox state and counteracts the effect of ROS (D’Onofrio et al., 2018). As recently revealed, in response to oxidative stress, SIRT6 is phosphorylated by c-Jun N-terminal kinase (JNK) at residue serine10 and that this modification is necessary for efficient recruitment of poly (ADP-ribose) polymerase 1 (PARP1) to DNA break sites and for efficient repair of DSBs (Van Meter et al., 2016). Moreover, it provides the relationship between oxidative stress and DNA repair that is critical for hormetic response and age-related diseases. Furthermore, SIRT6-deficient human mesenchymal stem cells (hMSCs) exhibited accelerated functional decay, a feature predominately characterized by dysregulated redox metabolism and increased sensitivity to the oxidative stress. In addition, SIRT6 could help assemble nuclear factor erythroid 2-related factor 2 (Nrf2)-RNA polymerase II transcription complex, which was required for the transactivation of Nrf2-regulated antioxidant genes (Pan et al., 2016). SIRT6 has been shown to suppress oxidative stress in the ischemic brain and non-alcoholic fatty liver via regulation of Nrf2 (Ka et al., 2017; Zhang W. et al., 2017).

Nrf2 is an evolutionarily conserved redox-sensitive transcription factor that coordinates antioxidant responses, including enzymes that up-regulate detoxification and repair macromolecular damage induced by ROS (Suh et al., 2004; Ungvari et al., 2011a, b, c). It binds to the antioxidant response elements (AREs) and activates the transcription of many antioxidant genes, including gluthatione S-transferases (GSTs), heme oxygenase 1 (HO-1), NAD(P)H: quinone oxidoreductase 1 (NQO1), thioredoxin, thioredoxin reductase, as well as proteins involved in scavenging reactive oxygen species (ROS) (Kovac et al., 2015) and glutathione (GSH) biosynthesis and regeneration (Gorrini et al., 2013; Rezazadeh et al., 2019). More importantly, recent advances have identified that the decline in Nrf2-ARE activity is observed in aged cells, which account for that SIRT6 mediated deacetylation of H3K56 is a crucial event safeguarding age-related cells from oxidative stress-associated functional decay (Bailey-Downs et al., 2012; Valcarcel-Ares et al., 2012; Pan et al., 2016). Interestingly, another result indicated that SIRT6 mono-ADP-ribosylation of BAF170, a subunit of BRG/BRM associated factor (BAF) chromatin remodeling complex, is required for activation of a subset Nrf2 responsive genes upon oxidative stress (Rezazadeh et al., 2019). Anyway, these findings showed that SIRT6 serves as an activator of Nrf2-dependent gene transcription. In cardiovascular studies, especially in ischemia/reperfusion injury and vascular endothelial dysfunction, Nrf2 pathway is the major target of SIRT6 to exert antioxidant effects.

In addition, as an adaptive response to the oxidative stress environment, metal transcription factor (MTF) has been previously shown to be the key transcription factor for the induction of metallothionein (Mt) to participate in antioxidant stress (Ghoshal and Jacob, 2001; Laity and Andrews, 2007). The antioxidant stress function of MT in heart prevents cardiomyocytes from diabetic cardiomyopathy and myocardial infarction (Gu et al., 2017; Xue et al., 2019). Recently study revealed that SIRT6 can promote the expression of Mt1 and Mt2. Indeed, both Mt1 and Mt2 promoters were activated by SIRT6. Moreover, SIRT6 can physically interact with MTF1 to have a synergistic effect on those Mt gene promoters (Kim et al., 2019).

Overall, SIRT6 is involved in the regulation of oxidative stress in a variety of tissue cells. And a series of studies have provided compelling evidence demonstrating the pathogenic effect of oxidative stress in CVDs (Zhao et al., 2015; Förstermann et al., 2017; van der Pol et al., 2019). Therefore, targeting SIRT6 to inhibit the generation of ROS and promote the activation of antioxidants represent reasonable therapeutic strategies for CVDs in the future.

### SIRT6 and Inflammation

Inflammation is a complex biophysical response of the body to pathogen infection and tissue damage. Although acute inflammation was considered protective, chronic inflammation was linked to numerous diseases (Xiao et al., 2012). For instance, the occurrence of human aging-related diseases is related to chronic low-grade inflammation, which is characterized by increased levels of circulating IL-6 and C-reactive protein (CRP) (Ferrucci et al., 2005; Wikby et al., 2006). A considerable number of elderly people showed the activation of inflammatory bodies and elevated levels of IL-1 β, which are associated with the risk of chronic aging diseases (Franceschi and Campisi, 2014; Furman et al., 2017). This phenomenon has also been confirmed in elderly rodents and primates, where pro-inflammatory changes have occurred in gene expression profiles of vascular endothelial cells and smooth muscle cells, including upregulated expression of inflammatory cytokines [such as IL-6, IL-1 β, tumor necrosis factor-α (TNF-α)], chemokines, adhesion molecules, inducible nitric oxide synthase and other pro-inflammatory mediators. Moreover, it increases the risk of CVDs, including atherosclerotic visceral diseases (Csiszar et al., 2002, 2003, 2004; Ungvari et al., 2007; Song et al., 2012). In addition, according to the data and the hypotheses presented in the study (Ferrucci and Fabbri, 2018), modulating inflammation is a promising approach not only to prevent CVD but also to slow the decline of health that occurs with aging.

Within the past few years, sirtuins have been identified as novel regulators of the immune system (Yang et al., 2007; Csiszar et al., 2008; Yoshizaki et al., 2009), and several studies show that SIRT6 can suppress inflammation in different tissues (Kawahara et al., 2009, 2011; Zhang N. et al., 2016). One of the master regulators of both adaptive and innate immunity is the NF-κB, which forms complexes with many other proteins, including Rel family members (RelA/p65, c-Rel, and RelB). The NF-κB complexes can translocate from the cytoplasm into the nucleus to trigger expression of target genes that are largely pro-inflammatory. It has been demonstrated that SIRT6, as a potent inhibitor of the NF-κB system, providing a mechanistic link between inflammation and aging (Kawahara et al., 2009; Zhang N. et al., 2016). A study revealed that SIRT6 promoted microRNA-21 expression, this reduced the expression of TGF-β2 and IL-1α and decreased the production of type I collagen and fibroblast proliferation (Fan et al., 2016). Besides, it has been found that SIRT6 restrained TGF-β signaling by deacetylation of H3K9 and H3K56. SIRT6 haploinsufficiency was sufficient for enhancing myofibroblast generation, leading to multiorgan fibrosis and cardiac dysfunction in mice during aging (Maity et al., 2020). Furthermore, recent report unveiled that overexpression of SIRT6 blocked the expression of NF-κB downstream regulators, such as interleukin (IL)-1β, IL-6, and matrix metalloproteinase 9 (MMP-9), all of which promoted fibroblast differentiation in TAC-induced cardiac fibrosis (Zhang et al., 2019).

In mouse liver, SIRT6 deacetylates H3K9 on the promoters of pro-inflammatory gene IL-6 and monocyte chemoattractant protein MCP-1 by inhibiting the transcriptional activity of c-JUN. After SIRT6 gene knockout, the expression of pro-inflammatory cytokines IL-1 β, IL-6, and TNF-α was upregulated significantly, while anti-inflammatory cytokines IL-10 was significantly down-regulated, causing chronic inflammation and fibrosis of the liver (Xiao et al., 2012; Kim et al., 2019). In addition, studies have found that SIRT6 also promotes the production and secretion of inflammatory cytokines (Van Gool et al., 2009; Bauer et al., 2012; Jiang et al., 2013, 2016), leading to chronic inflammation, which is the basis of neuronal death in Parkinson’s disease and other neurodegenerative diseases (Nicholatos et al., 2018). Therefore, it is worthy of detailed investigation of the relationship between SIRT6 and other aging-related diseases. In mouse glomerular podocytes, SIRT6 inhibits the transcription of Notch1 and Notch4 signals via epigenetic regulation, lowers the expression of inflammatory cytokines IL-1 β, IL-6, and TNF-α, protects podocytes from inflammatory damage, and effectively reduces the occurrence of chronic proteinuria nephropathy (Liu et al., 2017). In the adventitia inflammation induced by TNF-α, SIRT6 attenuates vascular inflammation by inhibiting the phosphorylation of Akt signal and the expression of monocyte chemoattractant proteins MCP-1 and IL-6 (He Y. et al., 2017).

### SIRT6 and Autophagy

Autophagy refers to the cellular process of degradation and recycling of long-lived or damaged organelles and proteins. This includes microautophagy (invagination of lysosomal membrane), molecular chaperone-mediated autophagy (transport of soluble proteins to lysosomes through molecular chaperones and lysosomal membrane receptors) and macroautophagy (where impurities are swallowed by double-membrane autophagosomes before lysosome fusion) (Shirakabe et al., 2016; Delbridge et al., 2017; Nakamura and Yoshimori, 2018; Zhang Y. et al., 2018). Autophagy is initiated by class III phosphatidylinositol-3 kinase (PI-3K) and Beclin-1. Autophagosomes undergo prolongation, microtubule light chain-3 (LC3) recruitment, LC3 proteolysis (lipolysis) to form autophagy lysosomes (fusion of autophagosomes and lysosomes), which regulate cell survival and senescence. Accumulating evidence suggested that autophagy is an essential mechanism for maintenance of tissue homeostasis in the heart during the aging process (Levine and Kroemer, 2008; Rubinsztein et al., 2011; Gatica et al., 2015). Mitophagy is an autophagic response that definitely targets damaged mitochondria. It was critical for the bioenergetics of the cardiovascular system, and mitophagy disorder could develop cardiac dysfunction (Bravo-San Pedro et al., 2017; Nicolas-Avila et al., 2020). Numerous studies have indicated that activate autophagy increase the healthy lifespan of animals, a positive effect that is generally associated with decelerated cardiovascular senescence (Zaglia et al., 2014; Gong et al., 2015; Eisenberg et al., 2016).

The two primary regulatory signal mechanisms implicated in autophagy disorders include inhibition of AMP-dependent protein kinase (AMPK) activation and up-regulation of class I PI-3K/Akt signal, resulting in excessive activation of rapamycin target (mTOR) signal and autophagy disorder (Kennedy and Lamming, 2016). Evidence has emerged that SIRT6 plays critical roles in the process of controlling autophagic degradation (Ng and Tang, 2013). Autophagy could be a harmful process to accelerate aging in some conditions. In a study of human bronchial epithelial cells, it was found that SIRT6 protects human bronchial epithelial cells from senescence by inhibiting insulin-like growth factor signaling-induced autophagy and regulating mTOR signaling (Shao et al., 2016).

However, in cardiovascular cells, autophagy mainly acts as beneficial process to maintain cellular homeostasis and delay aging. In macrophage foam cell from atherosclerosis model, SIRT6 and key autophagy effectors (ATG5, LC3B, and LAMP1) had been observed significantly and the overexpression of SIRT6 markedly reduced foam cell formation by inducing autophagy. The silencing of the key autophagy initiation gene ATG5 reversed the autophagy-promoting effect of SIRT6 with an increase in foam cells, which implied an autophagy-dependent pathway of SIRT6 in protecting against atherosclerosis by reducing foam cell formation (He J. et al., 2017).

Moreover, it has been found that Isoproterenol (ISO)-caused cardiac hypertrophy accompanying with a significant decrease in autophagy activity in primary neonatal rat cardiomyocytes (NRCMs). SIRT6 overexpression enhanced autophagy in NRCMs, whereas knockdown of SIRT6 by RNA interference led to suppression of cardiomyocyte autophagy (Lu et al., 2016). In terms of mechanism, SIRT6 activates FOXO3-dependent autophagy by reducing the level of Akt protein and phosphorylation, thereby enhancing the formation of LC3-II and down-regulating the expression of p62 (Lu et al., 2016). These results are consistent with the previous study that SIRT6 inhibit the transcription of IGF/Akt pathway genes via H3K9 deacetylation, which contributes to suppression of cardiac hypertrophy (Sundaresan et al., 2012).

### SIRT6, Genome Stability, and DNA Damage Repair

During the progression of aging, DNA, RNA, and proteins are constantly subjected to chemical alterations that impair their function (Ou and Schumacher, 2018). However, the consequences of DNA damage are much more widespread as DNA contains the information for all RNA and proteins a cell produces. It was considered that thousands of damaging events occur each day in every single one of our cells (De Bont, 2004). Persistent DNA damage can block transcription and replication thus hampering cellular functionality and promoting cellular senescence (Ou and Schumacher, 2018). To counteract the destructive effect of these actions and to maintain genomic integrity, it triggers a DNA damage response (DDR), which ensures efficient repair of all types of damage, including individual DNA base lesions and breaks (Ciccia and Elledge, 2010). The defects in DNA damage response further increases the burden of DNA damage, blocks cell cycle progression and causes the senescence of organs (Niedernhofer et al., 2018). Both endogenous and exogenous factors trigger DNA damage. The endogenous factors are the products of normal cell metabolism, resulting in oxidation, nitrification, and alkylation of DNA (Niedernhofer et al., 2018). On the other hand, exogenous factors, including ionizing radiation, ultraviolet radiation, and alkylating agents trigger DNA single-strand or double-strand breaks, which further causes an increase of inflammatory cytokines and accelerates the aging of the body (Rodier et al., 2009). Many repair mechanisms exist to ensure that nearly all the daily DNA damage is repaired, including base excision repair (BER), nucleotide excision repair (NER), non-homologous end connection (NHEJ), and homologous recombination (HR). Besides, each pathway identifies and repairs specific types of DNA damage to address most DNA damage, but not all DNA damage. It has been discovered that SIRT6 binds closely to chromatin and is an NAD^+^-dependent deacetylase of H3K9 and H3K56. Histone deacetylation is related to chromatin conformational closure and decreased chromatin accessibility. Therefore, the discovery of this enzyme activity confirmed the role of SIRT6 in regulating the dynamic binding of DNA damage repair and chromatin and gene expression (Michishita et al., 2008, 2009; Yang et al., 2014).

DNA damage is linked to several human diseases, including cancer, neurodegeneration, aging and CVDs (Madabhushi et al., 2014; Ou and Schumacher, 2018; Nakada et al., 2019; Reisländer et al., 2020). In the early stage of DNA damage, SIRT6 recruited SNF2H (an ATP-dependent chromatin remodeling) to DNA double-stranded breakpoint (DSB), to prevent genomic instability via local deacetylation of H3K56, and effectively recruited repair factors 53BP1, BRCA1, and RPA for damage repair (Toiber et al., 2013). In mammalian cells subjected to oxidative stress, SIRT6 was recruited to the DSB to bind to poly (ADP ribose) polymerase 1 (PARP1) and stimulated its activation by catalyzing the ADP ribose glycosylation of the K521 residue of PARP1, thus promoting the repair of DNA damage by connecting with non-homologous ends and HR (Mao et al., 2011). However, overactivation of PARP depletes the level of NAD content (Bürkle, 2001; Jagtap and Szabo, 2005), which is essential for the activity of sirtuins. It is critical to limit the overactivation of PARP1 in the heart to optimize its cardioprotective effect (Liu et al., 2014).

Further, reports indicate that stress-activated protein kinase JNK phosphorylates SIRT6 on serine 10 to promote DNA double-strand break (DSB) repair in response to oxidative stress. This post-translational modification helps to mobilize SIRT6 to the DNA damage site, effectively recruit PARP1 to the DNA cleavage site and promote repair factors 53BP1 and NBS1 to repair effectively (Van Meter et al., 2016). Chromatin relaxation is a prerequisite for the repair of DNA damage. Recent studies suggest that during DNA damage, SIRT6 rapidly shifts to the site of DNA damage, interacts with chromatin remover CHD4, and recruits CHD4, to promote the repair of DNA damage caused by chromatin relaxation. Once the damage site is reached, CHD4 replaces heterochromatin protein 1 (HP1) in histone H3K9 trimethylation (H3K9me3), whereas CHD4-dependent chromatin relaxation and H3K9me3 competition for the release of HP1, in damaged chromatin, are both necessary for precise HR (Hou et al., 2020). When repairing DNA damage via homologous recombination pathway, SIRT6 binds to DSB excision protein CtIP (carboxyl-terminal binding protein acting protein) and deacetylate CtIP to promote terminal excision (Kaidi et al., 2010). Highly unstable genomes were found in patients with multiple myeloma. A high level of SIRT6 promotes the repair of Chk1 DNA damage by triggering ERK2/p90RSK signal inactivation and offering resistance to DNA damage. The deletion of the SIRT6 gene enhances the sensitivity to DNA damage (Cea et al., 2016). It has been found that SIRT6 will be located at the damage site of single-strand breaks in a PARP1-dependent manner, and downstream repair factors will be recruited to promote base excision and repair of BER. In addition, the efficiency of BER decreased significantly with the increase of age, and overexpression of SIRT6 in senescent cells could significantly improve the efficiency of BER (Xu et al., 2015).

### SIRT6 and Telomere Homeostasis

Mammalian telomeres are the terminal structures of chromosomes, which comprise TTAGGG tandem repeats and associated protein complexes (protegerins). This complex protects chromosomes from end-to-end fusion and degradation by forming a special tring-like structure to avoid the ends of chromosomes being identified as double-stranded DNA breaks (Griffith et al., 1999; de Lange, 2005; Palm and de Lange, 2008). With each round of cell division, telomeres become shorter and when the shortened telomeres reach the critical length, it would trigger a sustained DDR and cell senescence (Shay, 2016). To escape senescence, cells might active or up-regulate telomerase, a cellular reverse transcriptase that adds new DNA to telomeres at the end of chromosomes. However, most normal human cells lack the telomerase that maintains telomeres (Baur, 2001). Therefore, telomere length is widely considered as a marker of biological aging, although this parameter does not strictly satisfy the criteria of cell senescence by the American Federation for Aging Research (Mather et al., 2011; De Meyer et al., 2018). The most factors that can modulate telomere length are also cardiovascular risk factors. In clinical studies, an association between short leukocyte telomere length (LTL) and cardiovascular disorders, including atherosclerosis, myocardial infarction, heart failure, and hypertension, has been repeatedly shown (Nowak et al., 2002; Samani and van der Harst, 2008).

In human fibroblasts with specific knockout of the SIRT6 gene, an apparent end-to-end fusion of chromosomes and premature senescence were observed, which could be effectively reversed by ectopic expression of telomerase, suggesting that SIRT6 is implicated in maintaining telomere stability. The lack of SIRT6 caused the excessive acetylation of H3K9, thus leading to telomere dysfunction (Michishita et al., 2008). Furthermore, the function of SIRT6 in maintaining telomere to protect from senescence is also proved in other tissues including vascular smooth muscle cells (VSMCs) (Cardus et al., 2013; Grootaert et al., 2021).

The deficiency of SIRT6 not only causes telomere damage, but also destroys the closed chromatin environment near the telomere and triggers telomere position effect (TPE) dysfunction (Tennen et al., 2011). TPE refers to the epigenetic silencing of proximal telomere genes (Aparicio et al., 1991; Buck and Shore, 1995; Ng et al., 2002; Altaf et al., 2007) and the intensity of telomere silencing enhances with the increase of telomere length (Kyrion et al., 1993; Buck and Shore, 1995). It has been found that the depletion of SIRT6 in human cells elicits TPE dysfunction, while the restoration of SIRT6 expression is sufficient to reconstruct the silencing of the telomere gene (Tennen et al., 2011). Together, these findings establish new roles for SIRT6 in regulating an aging-associated epigenetic silencing process and provide new mechanistic insights into chromatin silencing at telomeres (Baur, 2001; Tennen et al., 2011; Robin et al., 2014).

In addition, telomere repeat binding factor 2 (TRF2), as a significant regulator of telomere integrity, it exerts telomere protection by blocking ATM signal and non-homologous terminal connection to (NHEJ) and promoting telomere replication (van Steensel et al., 1998; Denchi and de Lange, 2007; Ye et al., 2010). Early research confirmed that plaque VSMCs senescence associated with the loss of TRF2 that plays a critical role in process of atherosclerosis (Wang et al., 2015). Interestingly, recent study reveals a novel molecular mechanism that SIRT6 specifically interacts with TRF2 and promotes TRF2 degradation in response to DNA damage (Rizzo et al., 2017).

Taken together, we reviewed the implication of SIRT6 in maintaining the telomere stability. However, recent studies uncover a telomere-dependent control of sirtuins expression and raise the possibility of a feed forward loop whereby damaged telomeres decrease sirtuins expression, which could further impair telomere integrity, leading to a progressive deterioration (Amano et al., 2019). Telomere dysfunction and sirtuins repression, independently, are highly associated with susceptibility to CVDs, accelerated aging, and lifespan reduction, and these two pathways are closely intertwined and cooperate to drive disease.

## SIRT6 and Aging Related Cardiovascular Diseases

Mounting evidence indicates that activation of SIRT6 can have beneficial effects in CVDs, including atherosclerosis (Zhang Z. et al., 2016; Wang et al., 2020; Grootaert et al., 2021), cardiac hypertrophy (Sundaresan et al., 2012; Lu et al., 2016; Zhang X. et al., 2016), hypertension (Guo et al., 2019), and heart failure (Li et al., 2017; Table 1).

**TABLE 1:** SIRT6 is directly involved in the regulation of cardiovascular diseases.

**SIRT6-linked CVD**	**Targets**		**SIRT6-linked cellular function**	**Species**	**References**
Atherosclerosis	H3K9		Telomere homeostasis	Human/mouse	Grootaert et al., 2021
	H3K9/H3K56	NKG2D ligands			Zhang Z. et al., 2016
			Autophagy	Mouse	Wang et al., 2020
	Msr1			Mouse	Arsiwala et al., 2020
			Inflammation	Diabetic patients	Balestrieri et al., 2015
Cardiac fibrosis	H3K9/H3K56	TGF-β		Mouse	Maity et al., 2020
	AMPK-ACE2			Rats	Zhang Z. Z. et al., 2017
	NF-κB			Rats	Tian et al., 2015
Cardiac hypertrophy	H3K9	c-JUN IGF-AKT		Human/mouse	Sundaresan et al., 2012
			Autophagy	Rats	Lu et al., 2016
	NF-κB	PI3K/Akt		Rats	Shen et al., 2016
	STAT3			Rats	Zhang X. et al., 2016
	NF-κB			Rats	Yu et al., 2013
	NFATc4			Rats	Li et al., 2018
Cardiac glucose metabolism	FOXO1/PDK4			Mouse	Khan D. et al., 2018
Cardioprotection against apoptosis	TIP60-GATA4			Mouse	Peng et al., 2020
Cardioprotection against hypoxia	pAMPKα/NF-κB			Mouse	Maksin-Matveev et al., 2015
Heart failure			Telomere homeostasis	Mouse	Li et al., 2017
Hypertension	H3K9	Nkx3.2-GATA5		Mouse	Guo et al., 2019
Coronary artery disease	Two tagSNPs rs352493 and rs3760908 within SIRT6 Gene			Chinese Han population	Tang et al., 2016
Myocardial infarction	Two tagSNPs rs3760905 and rs4359565 within SIRT6 Gene		–	MI patients	Wang et al., 2016

It has been established as a significant factor and regulates essential molecular pathways in multiple pathological conditions. In addition, cardiocytes with SIRT6 specific knockout show accumulation of lactate, indicating compromised mitochondrial oxidation. The mechanism involves the activation of FOXO1-mediated transcription of PDK4 to modulate cardiac glucose metabolism (Khan D. et al., 2018). Furthermore, it is important for pancreatic beta cells to improve insulin secretion through the activation of SIRT6. Therefore, pharmacological activation of SIRT6 may be useful to enhance insulin secretion and it has potential for the development of effective drugs to treat diabetic cardiomyopathy (Xiong et al., 2016). When subjected to prolonged hypoxia, cardiomyocytes from transgenic mice with overexpression of SIRT6 showed the improved survival owing to the block of necrosis/apoptosis pathways (Maksin-Matveev et al., 2015).

### SIRT6 and Atherosclerosis

Atherosclerosis is the primary trigger of vascular diseases across the globe with ischemic heart disease being one of its major complications (Herrington et al., 2016). Several studies have shown that endothelial cell dysfunction, abnormal lipid metabolism, and other factors are implicated in the occurrence of atherosclerosis (Gimbrone and García-Cardeña, 2016; Musunuru and Kathiresan, 2016). Vascular smooth muscle cells (VSMCs) comprise a major cellular component of the atherosclerotic plaque. VSMCs in human atherosclerotic plaques are characterized by apoptosis, DNA damage, inflammation and an altered energy metabolism (Grootaert et al., 2018). Furthermore, VSMCs from human atherosclerotic plaques undergo senescence and it promotes atherosclerosis and plaque instability (Wang et al., 2015), while removal of senescent cells can reduce atherosclerosis (Childs et al., 2016). Recent study has demonstrated that SIRT6 protein (but not mRNA) expression is declined in VSMCs in human and mouse atherosclerotic plaques (Grootaert et al., 2021). Besides, VSMC-specific overexpression of SIRT6 restrains atherogenesis and decreases tissue markers of cell senescence and inflammation, dependent upon its deacetylase activity. This indicates that endogenous levels of SIRT6 is a critical regulator of VSMC senescence and reveals a therapeutic potential of SIRT6 in atherosclerosis.

#### SIRT6 and Endothelial Dysfunction

Early study has revealed that vascular endothelial maintains vascular tension, inhibits atherosclerosis, and forms a barrier to control the migration of various substances between blood vessels and tissues (Galley and Webster, 2004). It is an important locus of critical regulatory nodes to retain the homeostasis of the cardiovascular system. Endothelial cell dysfunction encompasses a constellation of various non-adaptive alterations in functional phenotype, which have important implications for the regulation of hemostasis and thrombosis, local vascular tone and redox balance, and the orchestration of acute and chronic inflammatory reactions within the arterial wall (Gimbrone and García-Cardeña, 2016). Therefore, it is significant to point out that endothelial cell dysfunction is involved in many disease processes, including atherosclerosis, pulmonary arterial hypertension and sepsis (Gimbrone and García-Cardeña, 2016; Joffre et al., 2020; Evans et al., 2021). Here we focus on the involvement of SIRT6 in atherosclerosis and endothelial cell dysfunction.

In the process of atherosclerosis, there exist several factors of endothelial cell dysfunction, including endothelial vasodilation damage, endothelial cell injury and repair disorder, abnormal expression of endothelial adhesion molecules as well as cytokines. Endothelial cell dysfunction manifested in lesion-prone areas of the arterial vasculature results in the earliest detectable changes in the life history of an atherosclerotic lesion (Stary, 2000; Virmani et al., 2000).

SIRT6 is expressed in endothelial-rich tissues including the aorta, lung, and brain. In SIRT6 gene knockout mice and endothelium-specific knockout mice, endothelium-dependent vasodilation of aorta to acetylcholine (Ach) was significantly impaired (Xu et al., 2017). To prevent atherosclerosis, maintain the health of endothelial cells, and slow down the aging process of endothelial cells, it is significantly vital to repair damaged endothelial cells (Lappas, 2012). SIRT6 protects endothelial cells from telomere and DNA damage, prevents premature senility, and maintains the ability of cell replication and angiogenesis *in vitro*, all of which are known to inhibit the development of endothelial dysfunction (Cardus et al., 2013). Endothelial cell adhesion molecules, including vascular cell adhesion molecule (VCAM-1), play an important role in atherosclerosis by promoting the adhesion of monocytes to inflammatory endothelium (Libby et al., 2009, 2011). The role of SIRT6 in monocyte adhesion to endothelial cells was evaluated by transfecting SIRT6 into human umbilical vein endothelial cells or interfering with its expression. It has been shown that SIRT6 inhibited monocyte adhesion by lowering the expression of VCAM-1 in endothelial cells induced by TNF-α (Xu et al., 2017).

Also, the abnormal expression of endothelial inflammatory factors regulated by SIRT6 is implicated in the formation of atherosclerosis. Damaged vascular cells (endothelium and smooth muscle) are active in secreting cytokines including IL-1, monocyte chemoattractant protein-1 (MCP-1) and granulocyte-monocyte stimulating factor (GM-CSF). These cytokines produce local intercellular autocrine and paracrine signal rings in the vascular wall to promote the progression of atherosclerosis (Pober and Sessa, 2007; Gimbrone and García-Cardeña, 2016). In the progression of atherogenesis, NF-κB signal tends to play a central role in the pro-inflammatory activation of endothelial cells by regulating the expression of many downstream molecules such as VCAM-1 and MCP-1 (Collins and Cybulsky, 2001). Noteworthy, SIRT6 interacts with NF-κB RELA subunits and deacetylates H3K9 on the promoter of NF-κB target gene to attenuate NF-κB signal (Kawahara et al., 2009). Furthermore, recent studies found that the activation of NF-κB might trigger fundamental changes in the chromatin structure of endothelial cells via the formation of super-enhancer complexes, hence, regulating the epigenetic level of the phenotype of pro-inflammatory endothelial cells in the process of atherosclerosis (Brown et al., 2014).

Recent reports argued that the epigenetic regulation of NKG2D ligands is also involved in atherosclerosis of SIRT6 heterozygous mice. The down-regulation of SIRT6 up-regulates the expression of NKG2D ligand and causes an increased expression of inflammatory cytokines, which could be nearly completely blocked by NKG2D ligand inhibition (Zhang Z. et al., 2016). Notably, Tumor necrosis factor superfamily member 4 (TNFSF4) is a gene affecting atherosclerosis susceptibility and encodes OX40 ligand. SIRT6 inhibits atherosclerosis by deacetylating H3K9 on the promoter of the TNFSF4 gene (Wang et al., 2005). Studies have found trace cholesterol crystals (CCs) in atherosclerotic plaques, which represent one of the mechanisms causing endothelial dysfunction. In HUVECs, SIRT6 significantly promotes eNOS activity and down-regulates the expression of intercellular adhesion molecules (ICAM-1) and VCAM1 by activating Nrf2, thereby alleviating endothelial dysfunction induced by CCs (Jin et al., 2020). In SIRT6 knockout mice, atherosclerotic plaque was enlarged, plaque vulnerability was enhanced, and the expression of ICAM-1 in aortic endothelial cells was significantly up-regulated, implying that SIRT6 is the primary negative regulator of endothelial dysfunction and atherosclerotic development (Liu et al., 2016).

#### SIRT6 and Lipid Metabolism

Sources of evidence from clinical studies suggest that low-density lipoprotein-cholesterol causes atherosclerosis-related CVDs (Ference et al., 2017). As such, regulating homeostasis of LDL- cholesterol is significantly critical to body health. Further, additional evidence asserts that proprotein converting enzyme subtilin/Kexin 9 (PCSK9) binds to liver low-density lipoprotein receptor (LDLR) and promotes its degradation in lysosomes, causing a decrease in LDL uptake and an increase in LDL cholesterol concentration (Bergeron et al., 2015). Besides, overexpression of the SIRT6 gene could reduce the level of LDL- cholesterol in hepatocytes of mice fed with a high-fat diet. It has been confirmed that SIRT6 could be recruited by FOXO3 to the promoter region of the PCSK9 gene and inhibit its expression through deacetylation of H3K9 and H3K56, thereby reducing the level of LDL- cholesterol. SIRT6 deficiency can lead to an upregulated expression of the PCSK9 gene and an increase of LDL-cholesterol (Tao et al., 2013a). Also, sterol regulatory element-binding protein (SREBP)-2, which controls the expression of cholesterol biosynthesis rate-limiting enzyme HMG-CoA, is a vital regulator of cholesterol biosynthesis. At the mechanism level, SIRT6 was also recruited to the (SREBP)-2 gene promoter by FOXO3 to inhibit its expression and reduce cholesterol biosynthesis via deacetylation of H3K9 and H3K56 (Tao et al., 2013b).

Additionally, the formation of macrophage foam cells is a typical pathological change of early atherosclerotic (AS), which is primarily due to the imbalance between cholesterol inflow and efflux in mononuclear macrophages and the accumulation of cholesterol ester (CE) in cytoplasmic lipid droplets (LDs) (Moore and Tabas, 2011). Moreover, oxidized low-density lipoprotein cholesterol (ox-LDL), which binds to scavenger receptor (Sr) and accumulates in the cytoplasm has been reported to play a pathogenic role in the occurrence and development of AS (Mitra et al., 2011). Under the condition of ox-LDL, SIRT6 inhibits the expression of miR-33 (an mRNA that negatively regulates ABCA1 and ABCG1), promotes autophagy and cholesterol efflux, and reduces the formation of macrophage foam cells, thereby delaying the progress of AS. SIRT6 gene knockout promotes the formation of macrophage foam cells, hence promoting the formation of atherosclerosis (He J. et al., 2017). Generally, these findings suggest that SIRT6 plays a vital role in low-density lipoprotein cholesterol metabolism, potentially counteracting the formation of atherosclerosis.

### SIRT6, Myocardial Hypertrophy, and Heart Failure

After birth, cardiomyocytes withdraw from the cell cycle and become terminally differentiated cells. In adult hearts, compensatory cardiac hypertrophy develops into cardiac hypertrophy by increasing the size of individual cardiomyocytes rather than the number of cardiomyocytes to cope with increased workload. This compensatory mechanism is accompanied by an increase in the size of cardiomyocytes, and the imbalance of fetal genetic programming as well as an increase of protein synthesis (Rohini et al., 2010). Hypertrophy is initially an adaptive response to physiological and pathological stimuli, however, pathological hypertrophy usually progresses to heart failure under the regulation of different cellular signaling pathways (Nakamura and Sadoshima, 2018). The incidence of cardiac hypertrophy sharply increases with age, implying that aging-related mechanisms might play a key role in the molecular regulation of myocardial hypertrophy (Lakatta and Levy, 2003). Reports have confirmed that SIRT6 plays a negative regulatory role in cardiac hypertrophy. SIRT6 knockout mice have cardiac hypertrophy and heart failure, while SIRT6 transgenic mice are not influenced by hypertrophy (Sundaresan et al., 2012). In addition to myocardial hypertrophy, studies have shown that the progression of heart failure is also related to extensive fibrosis, abnormal activation of insulin-like growth factor (IGF)-Akt signal, cardiac hyperstress mediated by β-adrenoceptor, and damage of autophagy (Tian et al., 2015; Lu et al., 2016; Zhang W. et al., 2016).

In the model of hypertrophic cardiomyocytes induced by angiotensin II and coarctation of the abdominal aorta, SIRT6 inhibits the transcriptional activity of NF-κB by deacetylating H3K9, thereby inhibiting cardiac hypertrophy (Yu et al., 2013). In addition, in cardiac fibroblasts stimulated by angiotensin II and rat myocardium treated with coarctation of the abdominal aorta, it was further confirmed that SIRT6 inhibited the transcriptional activity of NF-κB via deacetylation of H3K9, and inhibited cardiac fibroblasts differentiation into myofibroblasts, thus inhibiting cardiac fibrosis. In SIRT6 knockout cardiac fibroblasts, extracellular matrix deposition and α-SMA increase promote the transformation into myofibroblasts and trigger extensive cardiac fibrosis (Tian et al., 2015).

Additionally, the level of intracellular NAD plays a crucial role in cardiomyocyte hypertrophy. The expression of Nmnat2 (central enzyme of NAD biosynthesis) is down-regulated in hypertrophic cardiomyocytes induced by angiotensin II and constriction of abdominal aorta. Overexpression of Nmnat2 promotes the activation of SIRT6 and blocks angiotensin II-induced cardiac hypertrophy (Cai et al., 2012). Increasing evidence reveals that overactivation of PARP-1 plays a key role in the pathogenesis of cardiac hypertrophy and heart failure. Nonetheless, excessive activation of PARP-1 depletes its substrate NAD and causes cell death. Being a new PARP-1 inhibitor, AG-690/11026014 protects cardiomyocytes from angiotensin II-induced hypertrophy by restoring the NAD level and SIRT6 activity (Liu et al., 2014). The abnormal activation of insulin-like growth factor (IGF)-Akt signal is closely linked to the occurrence and development of numerous diseases including heart failure. Studies on the hearts of mice confirmed that SIRT6 inhibits the activation of the IGF-Akt signal by inhibiting c-Jun transcriptional activity and deacetylation in H3K9, thereby blocking cardiac hypertrophy. Nonetheless, SIRT6 knocked-out mice promotes the over-activation of multiple IGF signal-related genes, leading to cardiac hypertrophy and heart failure (Sundaresan et al., 2012).

Elsewhere, studies found that the activation of signal transducer and activator of transcription 3 (STAT3) is critical in β-adrenergic receptor-mediated pathological remodeling and heart failure. In phenylephrine (PE)-induced hypertrophic cardiomyocyte model and isoproterenol (ISO) induced hypertrophic rat model, the mRNA and protein expression of STAT3 and phosphorylation level (P-STAT3) was significantly upregulated, while the hypertrophic biomarkers including ANF and BNP increased. In contrast, the deacetylase activity of SIRT6 decreased, while the effect of PE-induced hypertrophy could be eliminated by overexpression of the SIRT6 gene. Similarly, the up-regulation of ANP and BNP caused by SIRT6 gene knockout can be reversed by silencing of STAT3. Besides, SIRT6 has been suggested to protect cardiomyocytes from hypertrophy by preventing PE-induced STAT3 activation (Zhang X. et al., 2016).

In the heart, autophagy promotes survival primarily by clearing misfolded protein aggregates and damaged organelles accumulated in cardiomyocytes during cellular stress and nutritional deprivation. While long-term up-regulation of autophagy triggers self-destruction and leads to heart failure (De Meyer and Martinet, 2009). After treatment of primary neonatal rat cardiomyocytes with ISO, apparent hypertrophy and autophagy damage were observed. Also, it was confirmed that SIRT6 could protect cardiomyocytes from hypertrophy by inhibiting the Akt signal, thus, promoting the activation of FOXO3 transcription factor, and enhancing autophagy (Lu et al., 2016).

In addition, compensatory hypertrophy of cardiomyocytes is related to the increase of protein synthesis. One of the master regulators of protein synthesis inside the cell is the nutrient and energy sensor kinase mechanistic target of rapamycin (mTOR) (Laplante and Sabatini, 2009; Saxton and Sabatini, 2017). It has been found that SIRT6 acts as a key regulator of cellular protein synthesis by transcriptionally regulating the mTOR signaling in partnership with the transcription factor Sp1, and the whole process independent of its deacetylase activity (Ravi et al., 2019). Besides, in the hypertrophic heart induced by ISO, the expression of SIRT6 was down-regulated, while the inhibition of mTOR restored cardiac function in muscle-specific SIRT6 knockout mice, which spontaneously developed into cardiac hypertrophy (Saxton and Sabatini, 2017; Ravi et al., 2019). Taken together these data establish a critical connection between SIRT6, mTOR signaling, protein synthesis and cardiac hypertrophy. It will contribute toward understanding and treating diverse pathologies associated with aging.

## Clinical Application Prospect in Aging and CVDs

Given the advantaged effects of SIRT6 in regulating cell senescence and CVDs, targeted activation of SIRT6 and its downstream mechanism signals will be a potential way of delaying aging and treating CVDs. Here, we mainly discuss the activators of SIRT6 in the existing or potential clinical application in aging and CVDs.

Caloric restriction (CR), the significant decrease in calorie intake, is a strategy for improving health and increasing lifespan (Madeo et al., 2019). It has been shown to improve heart function, suppress markers of inflammation and reduce the risk of CVDs and diabetes in humans (Caristia et al., 2020; Kirkham et al., 2020). The beneficial effects of CR occur through an extreme wide range of molecular mechanisms, largely overlapping with epigenetic factors like sirtuins (Gensous et al., 2019) and the promotion of autophagy process (Abdellatif et al., 2018). However, CR has been shown to increase risk of diminishing muscle strength, aerobic capacity, and bone mineral density (Mattison et al., 2012). Therefore, proper exercise in addition to a CR diet is crucial. Resent study found that calorie restriction and physical exercise effectively regulate the activity of sirtuins. For instance, exercise training can effectively regulate the activity of SIRT6 in the skeletal muscle of aged rats and delay the aging process (Koltai et al., 2010). Moreover, caloric restriction significantly improved the renal insufficiency of aged rats, enhanced the expression of SIRT6 and inhibited the transduction of NF-κB signal (Koltai et al., 2010; Zhang N. et al., 2016). These findings suggested that CR was a beneficial life habit that can delay the aging process by regulating SIRT6, which is worthy of the attention of patients with aging-related diseases.

So far, the compounds that can specifically regulate the activity of SIRT6 in CVDs are still limited. The Chinese herbal medicine, icariin, widely used in eastern countries to treat specific age-related diseases, including CVDs and the improvement of neurological function, has been proved to be an activator of SIRT6. In an *in vitro* cell model, it was discovered that 10^–16^–10^–8^mol/L icariin could effectively activate the expression of SIRT6 protein and delay cell senescence by inhibiting NF-κB signal transduction (inhibiting the expression of TNF-α, ICAM-1, IL-2, and IL-6). In the future, we need to supplement the clinical research of icariin in the treatment of CVDs (Chen et al., 2015).

As a water-soluble natural amino acid, ergothioneine (Egt) exists widely in animals and plants. It accumulates a high concentration in some tissues via food chain intake (Halliwell et al., 2018). Several lines of evidence show that it has the effect of anti-oxidation and anti-cell aging, including the protective effect on CVDs and chronic inflammatory injury (D’Onofrio et al., 2016; Servillo et al., 2017). Moreover, it has been found that Egt inhibits the aging process by activating the expression of SIRT1 and SIRT6 protein in endothelial cells, thus reducing the production of ROS and suppressing the downstream NF-κB pathway (Tang et al., 2015). Nonetheless, so far, while acknowledging the absence of toxicity in the range of millimoles of intracellular concentration, the number of clinical studies to evaluate the efficacy and safety of dietary supplementation of Egt is still limited. As such, enriching additional studies on the treatment of CVDs with large samples of Egt is critical.

Other activators of SIRT6 have the biological function of anti-cancer, while the potential effect of these activators in CVDs needs further studies. UBCS039 directly binds to SIRT6 at the hydrophobic pocket and induces H3K9 and H3K56 deacetylation in breast cancer and colorectal cancer cells (Iachettini et al., 2018). Quinoline-4-carboxamides is an excellent selective SIRT6 activator with the function of antiviability and antiproliferation activities in pancreatic ductal adenocarcinoma (PDAC) cells through decreased acetylation leved of H3K9, H3K18, and H3K56 (Chen et al., 2020). Moreover, recent studies have identified allosteric SIRT6 activators, MDL-800, MDL-801, and MDL-811, which bound to the surface allosteric site of SIRT6 and activate SIRT6 deacetylation by promoting the binding affinity of acetylated substrates to cofactor. They also exert a tumor suppressor effect by reducing the acetylation level of H3K9 and H3K56, thus leading to cell cycle arrest in hepatocellular carcinoma, colorectal cancer and non-small cell lung cancer (Huang et al., 2018). We predict that MDL-800 could reduce the Ischemia reperfusion injury in cardiomyocytes, direct evidence of the function of MDL-800 in heart has yet to be reported. Given the importance of deacetylation of histones in CVDs more in-depth studies on these SIRT6 activators in CVDs are essential in the future.

In conclusion, existing studies have shown that SIRT6 is an endogenous regulatory molecule for the inhibition of cell senescence and the prevention and treatment of CVDs. Specifically, SIRT6 performs its different cellular functions via acetyl and long-chain fatty acyl deacetylation as well as ADP- ribosylation, maintains genomic stability by regulating DNA repair and telomere homeostasis. Moreover, it inhibits cell aging by regulating oxidative stress and inflammatory autophagy, plays a profound role in CVDs by regulating triglyceride synthesis and (LDL) cholesterol homeostasis. Therefore, the regulation of SIRT6 activity might influence various human diseases and prolong life. Nonetheless, the molecular mechanism of regulating the activity and function of SIRT6 in the process of anti-aging as well as prevention and treatment of CVDs warrants deeper understanding. With a better understanding of biology, novel clinical treatments can also be designed to activate SIRT6. Additional biological targets are likely to be discovered in the future, laying a basis for understanding the importance of SIRT6 in human aging and CVDs.

## Author Contributions

All authors conceptualized and wrote the manuscript.

## Conflict of Interest

The authors declare that the research was conducted in the absence of any commercial or financial relationships that could be construed as a potential conflict of interest.

## References

[B1] Abdellatif M. Sedej S. Carmona-Gutierrez D. Madeo F. Kroemer G. (2018). Autophagy in cardiovascular aging. *Circ. Res.* 123 803–824. 10.1161/CIRCRESAHA.118.312208 30355077

[B2] Acin-Perez R. Lechuga-Vieco A. V. Del Mar Muñoz M. Nieto-Arellano R. Torroja C. Sánchez-Cabo F. (2018). Ablation of the stress protease OMA1 protects against heart failure in mice. *Sci. Transl. Med.* 10:eaan4935. 10.1126/scitranslmed.aan4935 29593106

[B3] Altaf M. Utley R. T. Lacoste N. Tan S. Briggs S. D. Côté J. (2007). Interplay of chromatin modifiers on a short basic patch of histone H4 tail defines the boundary of telomeric heterochromatin. *Mol. Cell* 28 1002–1014. 10.1016/j.molcel.2007.12.002 18158898 PMC2610362

[B4] Amano H. Chaudhury A. Rodriguez-Aguayo C. Lu L. Akhanov V. Catic A. (2019). Telomere dysfunction induces sirtuin repression that drives telomere-dependent disease. *Cell Metab.* 29 1274–1290.e9. 10.1016/j.cmet.2019.03.001 30930169 PMC6657508

[B5] Aparicio O. M. Billington B. L. Gottschling D. E. (1991). Modifiers of position effect are shared between telomeric and silent mating-type loci in *S. cerevisiae*. *Cell* 66 1279–1287. 10.1016/0092-8674(91)90049-5 1913809

[B6] Arsiwala T. Pahla J. van Tits L. J. Bisceglie L. Gaul D. S. Costantino S. (2020). Sirt6 deletion in bone marrow-derived cells increases atherosclerosis – central role of macrophage scavenger receptor 1. *J. Mol. Cell. Cardiol.* 139 24–32. 10.1016/j.yjmcc.2020.01.002 31972266

[B7] Bailey-Downs L. C. Mitschelen M. Sosnowska D. Toth P. Pinto J. T. Ballabh P. (2012). Liver-specific knockdown of IGF-1 decreases vascular oxidative stress resistance by impairing the Nrf2-dependent antioxidant response: a novel model of vascular aging. *J. Gerontol. Ser. A Biol. Sci. Med. Sci.* 67A 313–329. 10.1093/gerona/glr164 22021391 PMC3309870

[B8] Balestrieri M. L. Rizzo M. R. Barbieri M. Paolisso P. Onofrio D. N. Giovane A. (2015). Sirtuin 6 expression and inflammatory activity in diabetic atherosclerotic plaques: effects of incretin treatment. *Diabetes* 64 1395–1406. 10.2337/db14-1149 25325735

[B9] Bauer I. Grozio A. Lasiglie D. Basile G. Sturla L. Magnone M. (2012). The NAD+-dependent histone deacetylase SIRT6 promotes cytokine production and migration in pancreatic cancer cells by regulating Ca2+ responses. *J. Biol. Chem.* 287 40924–40937. 10.1074/jbc.M112.405837 23086953 PMC3510797

[B10] Baur J. A. (2001). Telomere position effect in human cells. *Science* 292 2075–2077. 10.1126/science.1062329 11408657

[B11] Bergeron N. Phan B. A. P. Ding Y. Fong A. Krauss R. M. (2015). Proprotein convertase subtilisin/Kexin type 9 inhibition. *Circulation* 132 1648–1666. 10.1161/CIRCULATIONAHA.115.016080 26503748

[B12] Bravo-San Pedro J. M. Kroemer G. Galluzzi L. (2017). Autophagy and mitophagy in cardiovascular disease. *Circ. Res.* 120 1812–1824. 10.1161/CIRCRESAHA.117.311082 28546358

[B13] Brown J. D. Lin C. Y. Duan Q. Griffin G. Federation A. J. Paranal R. M. (2014). NF-κB directs dynamic super enhancer formation in inflammation and Atherogenesis. *Mol. Cell* 56 219–231. 10.1016/j.molcel.2014.08.024 25263595 PMC4224636

[B14] Buck S. W. Shore D. (1995). Action of a RAP1 carboxy-terminal silencing domain reveals an underlying competition between HMR and telomeres in yeast. *Gene. Dev.* 9 370–384. 10.1101/gad.9.3.370 7867933

[B15] Bürkle A. (2001). Physiology and pathophysiology of poly(ADP-ribosyl)ation ^∗^. *Bioessays* 23 795–806. 10.1002/bies.1115 11536292

[B16] Burnett C. Valentini S. Cabreiro F. Goss M. Somogyvári M. Piper M. D. (2011). Absence of effects of Sir2 overexpression on lifespan in *C. elegans* and *Drosophila*. *Nature* 477 482–485. 10.1038/nature10296 21938067 PMC3188402

[B17] Cai Y. Yu S. Chen S. Pi R. Gao S. Li H. (2012). Nmnat2 protects cardiomyocytes from hypertrophy via activation of SIRT6. *FEBS Lett.* 586 866–874. 10.1016/j.febslet.2012.02.014 22449973

[B18] Cardus A. Uryga A. K. Walters G. Erusalimsky J. D. (2013). SIRT6 protects human endothelial cells from DNA damage, telomere dysfunction, and senescence. *Cardiovasc. Res.* 97 571–579. 10.1093/cvr/cvs352 23201774 PMC3567786

[B19] Caristia S. De Vito M. Sarro A. Leone A. Pecere A. Zibetti A. (2020). Is caloric restriction associated with better healthy aging outcomes? A systematic review and meta-analysis of randomized controlled trials. *Nutrients* 12:2290. 10.3390/nu12082290 32751664 PMC7468870

[B20] Cea M. Cagnetta A. Adamia S. Acharya C. Tai Y. Fulciniti M. (2016). Evidence for a role of the histone deacetylase SIRT6 in DNA damage response of multiple myeloma cells. *Blood* 127 1138–1150. 10.1182/blood-2015-06-649970 26675349 PMC4778164

[B21] Chen Q. Ames B. N. (1994). Senescence-like growth arrest induced by hydrogen peroxide in human diploid fibroblast F65 cells. *Proc. Natl. Acad. Sci. U.S.A.* 91 4130–4134. 10.1073/pnas.91.10.4130 8183882 PMC43738

[B22] Chen X. Sun W. Huang S. Zhang H. Lin G. Li H. (2020). Discovery of potent small-molecule SIRT6 activators: structure–activity relationship and anti-pancreatic ductal adenocarcinoma activity. *J. Med. Chem.* 63 10474–10495. 10.1021/acs.jmedchem.0c01183 32787077

[B23] Chen Y. Sun T. Wu J. Kalionis B. Zhang C. Yuan D. (2015). Icariin intervenes in cardiac inflammaging through upregulation of SIRT6 enzyme activity and inhibition of the NF-kappa B pathway. *Biomed Res. Int.* 2015 1–12. 10.1155/2015/895976 25688369 PMC4320867

[B24] Chiao Y. A. Rabinovitch P. S. (2015). The aging heart: figure 1. *Cold Spring Harb. Perspect. Med.* 5:a025148. 10.1101/cshperspect.a025148 26328932 PMC4561390

[B25] Childs B. G. Baker D. J. Wijshake T. Conover C. A. Campisi J. van Deursen J. M. (2016). Senescent intimal foam cells are deleterious at all stages of atherosclerosis. *Science* 354 472–477. 10.1126/science.aaf6659 27789842 PMC5112585

[B26] Ciccia A. Elledge S. J. (2010). The DNA damage response: making it safe to play with knives. *Mol. Cell* 40 179–204. 10.1016/j.molcel.2010.09.019 20965415 PMC2988877

[B27] Collins T. Cybulsky M. I. (2001). NF-κB: pivotal mediator or innocent bystander in atherogenesis? *J. Clin. Invest.* 107 255–264. 10.1172/JCI10373 11160146 PMC199202

[B28] Csiszar A. Labinskyy N. Podlutsky A. Kaminski P. M. Wolin M. S. Zhang C. (2008). Vasoprotective effects of resveratrol and SIRT1: attenuation of cigarette smoke-induced oxidative stress and proinflammatory phenotypic alterations. *Am. J. Physiol. Heart C* 294 H2721–H2735. 10.1152/ajpheart.00235.2008 18424637 PMC2551743

[B29] Csiszar A. Ungvari Z. Edwards J. G. Kaminski P. Wolin M. S. Koller A. (2002). Aging-induced phenotypic changes and oxidative stress impair coronary arteriolar function. *Circ. Res.* 90 1159–1166. 10.1161/01.res.0000020401.61826.ea 12065318

[B30] Csiszar A. Ungvari Z. Koller A. Edwards J. G. Kaley G. (2003). Aging-induced proinflammatory shift in cytokine expression profile in rat coronary arteries. *FASEB J.* 17 1183–1185. 10.1096/fj.02-1049fje 12709402

[B31] Csiszar A. Ungvari Z. Koller A. Edwards J. G. Kaley G. (2004). Proinflammatory phenotype of coronary arteries promotes endothelial apoptosis in aging. *Physiol. Genomics* 17 21–30. 10.1152/physiolgenomics.00136.2003 15020720

[B32] De Bont R. (2004). Endogenous DNA damage in humans: a review of quantitative data. *Mutagenesis* 19 169–185. 10.1093/mutage/geh025 15123782

[B33] de Lange T. (2005). Shelterin: the protein complex that shapes and safeguards human telomeres. *Gene. Dev.* 19 2100–2110. 10.1101/gad.1346005 16166375

[B34] De Meyer G. R. Y. Martinet W. (2009). Autophagy in the cardiovascular system. *Biochim. Biophys. Acta* 1793 1485–1495. 10.1016/j.bbamcr.2008.12.011 19152812

[B35] De Meyer T. Nawrot T. Bekaert S. De Buyzere M. L. Rietzschel E. R. Andrés V. (2018). Telomere length as cardiovascular aging biomarker. *J. Am. Coll. Cardiol.* 72 805–813. 10.1016/j.jacc.2018.06.014 30092957

[B36] Delbridge L. M. D. Mellor K. M. Taylor D. J. Gottlieb R. A. (2017). Myocardial stress and autophagy: mechanisms and potential therapies. *Nat. Rev. Cardiol.* 14 412–425. 10.1038/nrcardio.2017.35 28361977 PMC6245608

[B37] Denchi E. L. de Lange T. (2007). Protection of telomeres through independent control of ATM and ATR by TRF2 and POT1. *Nature* 448 1068–1071. 10.1038/nature06065 17687332

[B38] Desantis V. Lamanuzzi A. Vacca A. (2017). The role of SIRT6 in tumors. *Haematologica* 103 1–4. 10.3324/haematol.2017.182675 29290628 PMC5777184

[B39] D’Onofrio N. Servillo L. Balestrieri M. L. (2018). SIRT1 and SIRT6 signaling pathways in cardiovascular disease protection. *Antioxid. Redox Signal.* 28 711–732. 10.1089/ars.2017.7178 28661724 PMC5824538

[B40] D’Onofrio N. Servillo L. Giovane A. Casale R. Vitiello M. Marfella R. (2016). Ergothioneine oxidation in the protection against high-glucose induced endothelial senescence: involvement of SIRT1 and SIRT6. *Free Radic. Biol. Med.* 96 211–222. 10.1016/j.freeradbiomed.2016.04.013 27101740

[B41] Eisenberg T. Abdellatif M. Schroeder S. Primessnig U. Stekovic S. Pendl T. (2016). Cardioprotection and lifespan extension by the natural polyamine spermidine. *Nat. Med.* 22 1428–1438. 10.1038/nm.4222 27841876 PMC5806691

[B42] Evans C. E. Cober N. D. Dai Z. Stewart D. J. Zhao Y. (2021). Endothelial cells in the pathogenesis of pulmonary arterial hypertension. *Eur. Respir. J.* 10.1183/13993003.03957-2020 [Epub ahead of print]. 33509961 PMC8316496

[B43] Fan X. Chen J. Shi D. A. Jia J. He J. Li L. (2016). The role and mechanisms of action of SIRT6 in the suppression of postoperative epidural scar formation. *Int. J. Mol. Med.* 37 1337–1344. 10.3892/ijmm.2016.2522 26987016

[B44] Ference B. A. Ginsberg H. N. Graham I. Ray K. K. Packard C. J. Bruckert E. (2017). Low-density lipoproteins cause atherosclerotic cardiovascular disease. 1. Evidence from genetic, epidemiologic, and clinical studies. A consensus statement from the European Atherosclerosis Society Consensus Panel. *Eur. Heart J.* 38 2459–2472. 10.1093/eurheartj/ehx144 28444290 PMC5837225

[B45] Ferrucci L. Corsi A. Lauretani F. Bandinelli S. Bartali B. Taub D. D. (2005). The origins of age-related proinflammatory state. *Blood* 105 2294–2299. 10.1182/blood-2004-07-2599 15572589 PMC9828256

[B46] Ferrucci L. Fabbri E. (2018). Inflammageing: chronic inflammation in ageing, cardiovascular disease, and frailty. *Nat. Rev. Cardiol.* 15 505–522. 10.1038/s41569-018-0064-2 30065258 PMC6146930

[B47] Förstermann U. Xia N. Li H. (2017). Roles of vascular oxidative stress and nitric oxide in the pathogenesis of atherosclerosis. *Circ. Res.* 120 713–735. 10.1161/CIRCRESAHA.116.309326 28209797

[B48] Franceschi C. Campisi J. (2014). Chronic inflammation (Inflammaging) and its potential contribution to age-associated diseases. *J. Gerontol. A Biol. Sci. Med. Sci.* 69 S4–S9. 10.1093/gerona/glu057 24833586

[B49] Furman D. Chang J. Lartigue L. Bolen C. R. Haddad F. Gaudilliere B. (2017). Expression of specific inflammasome gene modules stratifies older individuals into two extreme clinical and immunological states. *Nat. Med.* 23 174–184. 10.1038/nm.4267 28092664 PMC5320935

[B50] Galley H. F. Webster N. R. (2004). Physiology of the endothelium. *Brit. J. Anaesth.* 93 105–113. 10.1093/bja/aeh163 15121728

[B51] Gatica D. Chiong M. Lavandero S. Klionsky D. J. (2015). Molecular mechanisms of autophagy in the cardiovascular system. *Circ. Res.* 116 456–467. 10.1161/CIRCRESAHA.114.303788 25634969 PMC4313620

[B52] Gensous N. Franceschi C. Santoro A. Milazzo M. Garagnani P. Bacalini M. G. (2019). The impact of caloric restriction on the epigenetic signatures of aging. *Int. J. Mol. Sci.* 20:2022. 10.3390/ijms20082022 31022953 PMC6515465

[B53] Ghoshal K. Jacob S. T. (2001). Regulation of metallothionein gene expression. *Prog. Nucleic Acid Res. Mol. Biol.* 66 357–384. 10.1016/s0079-6603(00)66034-8 11051769

[B54] Gimbrone M. A. García-Cardeña G. (2016). Endothelial cell dysfunction and the pathobiology of atherosclerosis. *Circ. Res.* 118 620–636. 10.1161/CIRCRESAHA.115.306301 26892962 PMC4762052

[B55] Gong G. Song M. Csordas G. Kelly D. P. Matkovich S. J. Dorn G. N. (2015). Parkin-mediated mitophagy directs perinatal cardiac metabolic maturation in mice. *Science* 350:aad2459. 10.1126/science.aad2459 26785495 PMC4747105

[B56] Gorrini C. Harris I. S. Mak T. W. (2013). Modulation of oxidative stress as an anticancer strategy. *Nat. Rev. Drug Discov.* 12 931–947. 10.1038/nrd4002 24287781

[B57] Griendling K. K. FitzGerald G. A. (2003). Oxidative stress and cardiovascular injury. *Circulation* 108 2034–2040. 10.1161/01.CIR.0000093661.90582.c4 14581381

[B58] Griffith J. D. Comeau L. Rosenfield S. Stansel R. M. Bianchi A. Moss H. (1999). Mammalian telomeres end in a large duplex loop. *Cell* 97 503–514. 10.1016/s0092-8674(00)80760-6 10338214

[B59] Grootaert M. Finigan A. Figg N. L. Uryga A. K. Bennett M. R. (2021). SIRT6 protects smooth muscle cells from senescence and reduces atherosclerosis. *Circ. Res.* 128 474–491. 10.1161/CIRCRESAHA.120.318353 33353368 PMC7899748

[B60] Grootaert M. O. J. Moulis M. Roth L. Martinet W. Vindis C. Bennett M. R. (2018). Vascular smooth muscle cell death, autophagy and senescence in atherosclerosis. *Cardiovasc. Res.* 114 622–634. 10.1093/cvr/cvy007 29360955

[B61] Gu J. Cheng Y. Wu H. Kong L. Wang S. Xu Z. (2017). Metallothionein is downstream of Nrf2 and partially mediates sulforaphane prevention of diabetic cardiomyopathy. *Diabetes* 66 529–542. 10.2337/db15-1274 27903744 PMC5248986

[B62] Guo J. Wang Z. Wu J. Liu M. Li M. Sun Y. (2019). Endothelial SIRT6 is vital to prevent hypertension and associated cardiorenal injury through targeting Nkx3.2-GATA5 signaling. *Circ. Res.* 124 1448–1461. 10.1161/CIRCRESAHA.118.314032 30894089

[B63] Haigis M. C. Sinclair D. A. (2010). Mammalian sirtuins: biological insights and disease relevance. *Annu. Rev. Pathol.* 5 253–295. 10.1146/annurev.pathol.4.110807.092250 20078221 PMC2866163

[B64] Halliwell B. Cheah I. K. Tang R. (2018). Ergothioneine – a diet-derived antioxidant with therapeutic potential. *FEBS Lett.* 592 3357–3366. 10.1002/1873-3468.13123 29851075

[B65] He J. Zhang G. Pang Q. Yu C. Xiong J. Zhu J. (2017). SIRT6 reduces macrophage foam cell formation by inducing autophagy and cholesterol efflux under ox-LDL condition. *FEBS J.* 284 1324–1337. 10.1111/febs.14055 28296196

[B66] He Y. Xiao Y. Yang X. Li Y. Wang B. Yao F. (2017). SIRT6 inhibits TNF-α-induced inflammation of vascular adventitial fibroblasts through ROS and Akt signaling pathway. *Exp. Cell Res.* 357 88–97. 10.1016/j.yexcr.2017.05.001 28477980

[B67] Herrington W. Lacey B. Sherliker P. Armitage J. Lewington S. (2016). Epidemiology of atherosclerosis and the potential to reduce the global burden of Atherothrombotic disease. *Circ. Res.* 118 535–546. 10.1161/CIRCRESAHA.115.307611 26892956

[B68] Hou T. Cao Z. Zhang J. Tang M. Tian Y. Li Y. (2020). SIRT6 coordinates with CHD4 to promote chromatin relaxation and DNA repair. *Nucleic Acids Res.* 48 2982–3000. 10.1093/nar/gkaa006 31970415 PMC7102973

[B69] Huang Z. Zhao J. Deng W. Chen Y. Shang J. Song K. (2018). Identification of a cellularly active SIRT6 allosteric activator. *Nat. Chem. Biol.* 14 1118–1126. 10.1038/s41589-018-0150-0 30374165

[B70] Iachettini S. Trisciuoglio D. Rotili D. Lucidi A. Salvati E. Zizza P. (2018). Pharmacological activation of SIRT6 triggers lethal autophagy in human cancer cells. *Cell Death Dis.* 9 996. 10.1038/s41419-018-1065-0 30250025 PMC6155207

[B71] Jagtap P. Szabo C. (2005). Poly(ADP-ribose) polymerase and the therapeutic effects of its inhibitors. *Nat. Rev. Drug Discov.* 4 421–440. 10.1038/nrd1718 15864271

[B72] Jiang H. Khan S. Wang Y. Charron G. He B. Sebastian C. (2013). SIRT6 regulates TNF-alpha secretion through hydrolysis of long-chain fatty acyl lysine. *Nature* 496 110–113. 10.1038/nature12038 23552949 PMC3635073

[B73] Jiang H. Zhang X. Lin H. (2016). Lysine fatty acylation promotes lysosomal targeting of TNF-alpha. *Sci. Rep.* 6:24371. 10.1038/srep24371 27079798 PMC4832147

[B74] Jin Z. Xiao Y. Yao F. Wang B. Zheng Z. Gao H. (2020). SIRT6 inhibits cholesterol crystal-induced vascular endothelial dysfunction via Nrf2 activation. *Exp. Cell Res.* 387:111744. 10.1016/j.yexcr.2019.111744 31759967

[B75] Joffre J. Hellman J. Ince C. Ait-Oufella H. (2020). Endothelial responses in sepsis. *Am. J. Respir. Crit. Care Med.* 202 361–370. 10.1164/rccm.201910-1911TR 32101446

[B76] Jung E. S. Choi H. Song H. Hwang Y. J. Kim A. Ryu H. (2016). p53-dependent SIRT6 expression protects Abeta42-induced DNA damage. *Sci. Rep.* 6:25628. 10.1038/srep25628 27156849 PMC4860716

[B77] Ka S. O. Bang I. H. Bae E. J. Park B. H. (2017). Hepatocyte-specific sirtuin 6 deletion predisposes to nonalcoholic steatohepatitis by up-regulation of Bach1, an Nrf2 repressor. *FASEB J.* 31 3999–4010. 10.1096/fj.201700098RR 28536120

[B78] Kaeberlein M. (2010). Lessons on longevity from budding yeast. *Nature* 464 513–519. 10.1038/nature08981 20336133 PMC3696189

[B79] Kaeberlein M. McVey M. Guarente L. (1999). The SIR2/3/4 complex and SIR2 alone promote longevity in Saccharomyces cerevisiae by two different mechanisms. *Genes Dev.* 13 2570–2580. 10.1101/gad.13.19.2570 10521401 PMC317077

[B80] Kaidi A. Weinert B. T. Choudhary C. Jackson S. P. (2010). Human SIRT6 promotes DNA end resection through CtIP deacetylation. *Science* 329 1348–1353. 10.1126/science.1192049 20829486 PMC3276839

[B81] Kaluski S. Portillo M. Besnard A. Stein D. Einav M. Zhong L. (2017). Neuroprotective functions for the histone deacetylase SIRT6. *Cell Rep.* 18 3052–3062. 10.1016/j.celrep.2017.03.008 28355558 PMC5389893

[B82] Kanfi Y. Naiman S. Amir G. Peshti V. Zinman G. Nahum L. (2012). The sirtuin SIRT6 regulates lifespan in male mice. *Nature* 483 218–221. 10.1038/nature10815 22367546

[B83] Kawahara T. L. Rapicavoli N. A. Wu A. R. Qu K. Quake S. R. Chang H. Y. (2011). Dynamic chromatin localization of Sirt6 shapes stress- and aging-related transcriptional networks. *PLoS Genet.* 7:e1002153. 10.1371/journal.pgen.1002153 21738489 PMC3128103

[B84] Kawahara T. L. A. Michishita E. Adler A. S. Damian M. Berber E. Lin M. (2009). SIRT6 links histone H3 Lysine 9 deacetylation to NF-κB-dependent gene expression and organismal life span. *Cell* 136 62–74. 10.1016/j.cell.2008.10.052 19135889 PMC2757125

[B85] Kennedy B. K. Lamming D. W. (2016). The mechanistic target of rapamycin: the grand ConducTOR of metabolism and aging. *Cell Metab.* 23 990–1003. 10.1016/j.cmet.2016.05.009 27304501 PMC4910876

[B86] Kenyon C. J. (2010). The genetics of ageing. *Nature* 464 504–512. 10.1038/nature08980 20336132

[B87] Khan D. Sarikhani M. Dasgupta S. Maniyadath B. Pandit A. S. Mishra S. (2018). SIRT6 deacetylase transcriptionally regulates glucose metabolism in heart. *J. Cell. Physiol.* 233 5478–5489. 10.1002/jcp.26434 29319170

[B88] Khan R. I. Nirzhor S. Akter R. (2018). A review of the recent advances made with SIRT6 and its implications on aging related processes, major human diseases, and possible therapeutic targets. *Biomolecules* 8:44. 10.3390/biom8030044 29966233 PMC6164879

[B89] Kim H. G. Huang M. Xin Y. Zhang Y. Zhang X. Wang G. (2019). The epigenetic regulator SIRT6 protects the liver from alcohol-induced tissue injury by reducing oxidative stress in mice. *J. Hepatol.* 71 960–969. 10.1016/j.jhep.2019.06.019 31295533 PMC6801027

[B90] Kirkham A. A. Beka V. Prado C. M. (2020). The effect of caloric restriction on blood pressure and cardiovascular function: a systematic review and meta-analysis of randomized controlled trials. *Clin. Nutr.* 40 728–739. 10.1016/j.clnu.2020.06.029 32675017

[B91] Koltai E. Szabo Z. Atalay M. Boldogh I. Naito H. Goto S. (2010). Exercise alters SIRT1, SIRT6, NAD and NAMPT levels in skeletal muscle of aged rats. *Mech. Ageing Dev.* 131 21–28. 10.1016/j.mad.2009.11.002 19913571 PMC2872991

[B92] Kovac S. Angelova P. R. Holmstrom K. M. Zhang Y. Dinkova-Kostova A. T. Abramov A. Y. (2015). Nrf2 regulates ROS production by mitochondria and NADPH oxidase. *Biochim. Biophys. Acta* 1850 794–801. 10.1016/j.bbagen.2014.11.021 25484314 PMC4471129

[B93] Kugel S. Mostoslavsky R. (2014). Chromatin and beyond: the multitasking roles for SIRT6. *Trends Biochem. Sci.* 39 72–81. 10.1016/j.tibs.2013.12.002 24438746 PMC3912268

[B94] Kyrion G. Liu K. Liu C. Lustig A. J. (1993). RAP1 and telomere structure regulate telomere position effects in *Saccharomyces cerevisiae*. *Genes Dev* 7 1146–1159. 10.1101/gad.7.7a.1146 8319907

[B95] Laity J. H. Andrews G. K. (2007). Understanding the mechanisms of zinc-sensing by metal-response element binding transcription factor-1 (MTF-1). *Arch. Biochem. Biophys.* 463 201–210. 10.1016/j.abb.2007.03.019 17462582

[B96] Lakatta E. G. Levy D. (2003). Arterial and cardiac aging: major shareholders in cardiovascular disease enterprises: Part II: the aging heart in health: links to heart disease. *Circulation* 107 346–354. 10.1161/01.cir.0000048893.62841.f7 12538439

[B97] Laplante M. Sabatini D. M. (2009). mTOR signaling at a glance. *J. Cell Sci.* 122 3589–3594. 10.1242/jcs.051011 19812304 PMC2758797

[B98] Lappas M. (2012). Anti-inflammatory properties of sirtuin 6 in human umbilical vein endothelial cells. *Mediat. Inflamm.* 2012 1–11. 10.1155/2012/597514 23132960 PMC3486624

[B99] Lasry A. Ben-Neriah Y. (2015). Senescence-associated inflammatory responses: aging and cancer perspectives. *Trends Immunol.* 36 217–228. 10.1016/j.it.2015.02.009 25801910

[B100] Levine B. Kroemer G. (2008). Autophagy in the pathogenesis of disease. *Cell* 132 27–42. 10.1016/j.cell.2007.12.018 18191218 PMC2696814

[B101] Li Y. Meng X. Wang W. Liu F. Hao Z. Yang Y. (2017). Cardioprotective effects of SIRT6 in a mouse model of transverse aortic constriction-induced heart failure. *Front. Physiol.* 8:394. 10.3389/fphys.2017.00394 28659816 PMC5468374

[B102] Li Z. Zhang X. Guo Z. Zhong Y. Wang P. Li J. (2018). SIRT6 suppresses NFATc4 expression and activation in cardiomyocyte hypertrophy. *Front. Pharmacol.* 9:1519. 10.3389/fphar.2018.01519 30670969 PMC6331469

[B103] Liao C. Kennedy B. K. (2012). Will the real aging Sirtuin please stand up? *Cell Res.* 22 1215–1217. 10.1038/cr.2012.62 22508266 PMC3411170

[B104] Liao C. Y. Kennedy B. K. (2014). Mouse models and aging: longevity and progeria. *Curr. Top. Dev. Biol.* 109 249–285. 10.1016/B978-0-12-397920-9.00003-2 24947239

[B105] Libby P. Ridker P. M. Hansson G. K. (2009). Inflammation in atherosclerosis. *J. Am. Coll. Cardiol.* 54 2129–2138. 10.1016/j.jacc.2009.09.009 19942084 PMC2834169

[B106] Libby P. Ridker P. M. Hansson G. K. (2011). Progress and challenges in translating the biology of atherosclerosis. *Nature* 473 317–325. 10.1038/nature10146 21593864

[B107] Liu M. Li Z. Chen G. Li Z. Wang L. Ye J. (2014). AG-690/11026014, a novel PARP-1 inhibitor, protects cardiomyocytes from AngII-induced hypertrophy. *Mol. Cell. Endocrinol.* 392 14–22. 10.1016/j.mce.2014.05.010 24859603

[B108] Liu M. Liang K. Zhen J. Zhou M. Wang X. Wang Z. (2017). Sirt6 deficiency exacerbates podocyte injury and proteinuria through targeting Notch signaling. *Nat. Commun.* 8:413. 10.1038/s41467-017-00498-4 28871079 PMC5583183

[B109] Liu Z. Wang J. Huang X. Li Z. Liu P. (2016). Deletion of sirtuin 6 accelerates endothelial dysfunction and atherosclerosis in apolipoprotein E-deficient mice. *Transl. Res.* 172 18–29.e2. 10.1016/j.trsl.2016.02.005 26924042

[B110] Lu J. Sun D. Liu Z. Li M. Hong H. Liu C. (2016). SIRT6 suppresses isoproterenol-induced cardiac hypertrophy through activation of autophagy. *Transl. Res.* 172 96–112.e6. 10.1016/j.trsl.2016.03.002 27016702

[B111] Madabhushi R. Pan L. Tsai L. H. (2014). DNA damage and its links to neurodegeneration. *Neuron* 83 266–282. 10.1016/j.neuron.2014.06.034 25033177 PMC5564444

[B112] Madeo F. Carmona-Gutierrez D. Hofer S. J. Kroemer G. (2019). Caloric restriction mimetics against age-associated disease: targets, mechanisms, and therapeutic potential. *Cell Metab.* 29 592–610. 10.1016/j.cmet.2019.01.018 30840912

[B113] Maity S. Muhamed J. Sarikhani M. Kumar S. Ahamed F. Spurthi K. M. (2020). Sirtuin 6 deficiency transcriptionally up-regulates TGF-beta signaling and induces fibrosis in mice. *J. Biol. Chem.* 295 415–434. 10.1074/jbc.RA118.007212 31744885 PMC6956532

[B114] Maksin-Matveev A. Kanfi Y. Hochhauser E. Isak A. Cohen H. Y. Shainberg A. (2015). Sirtuin 6 protects the heart from hypoxic damage. *Exp. Cell Res.* 330 81–90. 10.1016/j.yexcr.2014.07.013 25066211

[B115] Mao Z. Hine C. Tian X. Van Meter M. Au M. Vaidya A. (2011). SIRT6 Promotes DNA repair under stress by activating PARP1. *Science* 332 1443–1446. 10.1126/science.1202723 21680843 PMC5472447

[B116] Mather K. A. Jorm A. F. Parslow R. A. Christensen H. (2011). Is telomere length a biomarker of aging? A review. *J. Gerontol. A Biol. Sci. Med. Sci.* 66A 202–213. 10.1093/gerona/glq180 21030466

[B117] Mattison J. A. Roth G. S. Beasley T. M. Tilmont E. M. Handy A. M. Herbert R. L. (2012). Impact of caloric restriction on health and survival in rhesus monkeys from the NIA study. *Nature* 489 318–321. 10.1038/nature11432 22932268 PMC3832985

[B118] Michishita E. McCord R. A. Berber E. Kioi M. Padilla-Nash H. Damian M. (2008). SIRT6 is a histone H3 lysine 9 deacetylase that modulates telomeric chromatin. *Nature* 452 492–496. 10.1038/nature06736 18337721 PMC2646112

[B119] Michishita E. McCord R. A. Boxer L. D. Barber M. F. Hong T. Gozani O. (2009). Cell cycle-dependent deacetylation of telomeric histone H3 lysine K56 by human SIRT6. *Cell Cycle* 8 2664–2666. 10.4161/cc.8.16.9367 19625767 PMC4474138

[B120] Miller M. W. Sadeh N. (2014). Traumatic stress, oxidative stress and post-traumatic stress disorder: neurodegeneration and the accelerated-aging hypothesis. *Mol. Psychiatry* 19 1156–1162. 10.1038/mp.2014.111 25245500 PMC4211971

[B121] Mitra S. Deshmukh A. Sachdeva R. Lu J. Mehta J. L. (2011). Oxidized low-density lipoprotein and atherosclerosis implications in antioxidant therapy. *Am. J. Med. Sci.* 342 135–142. 10.1097/MAJ.0b013e318224a147 21747278

[B122] Moore K. J. Tabas I. (2011). Macrophages in the pathogenesis of atherosclerosis. *Cell* 145 341–355. 10.1016/j.cell.2011.04.005 21529710 PMC3111065

[B123] Mostoslavsky R. Chua K. F. Lombard D. B. Pang W. W. Fischer M. R. Gellon L. (2006). Genomic instability and aging-like phenotype in the absence of mammalian SIRT6. *Cell* 124 315–329. 10.1016/j.cell.2005.11.044 16439206

[B124] Musunuru K. Kathiresan S. (2016). Surprises from genetic analyses of lipid risk factors for atherosclerosis. *Circ. Res.* 118 579–585. 10.1161/CIRCRESAHA.115.306398 26892959 PMC4762058

[B125] Nakada Y. Nhi Nguyen N. U. Xiao F. Savla J. J. Lam N. T. Abdisalaam S. (2019). DNA damage response mediates pressure overload–induced cardiomyocyte hypertrophy. *Circulation* 139 1237–1239. 10.1161/CIRCULATIONAHA.118.034822 30802166 PMC6467068

[B126] Nakamura M. Sadoshima J. (2018). Mechanisms of physiological and pathological cardiac hypertrophy. *Nat. Rev. Cardiol.* 15 387–407. 10.1038/s41569-018-0007-y 29674714

[B127] Nakamura S. Yoshimori T. (2018). Autophagy and longevity. *Mol. Cells* 41 65–72. 10.14348/molcells.2018.2333 29370695 PMC5792715

[B128] Ng F. Tang B. L. (2013). Sirtuins’ modulation of autophagy. *J. Cell. Physiol.* 228 2262–2270. 10.1002/jcp.24399 23696314

[B129] Ng H. H. Feng Q. Wang H. Erdjument-Bromage H. Tempst P. Zhang Y. (2002). Lysine methylation within the globular domain of histone H3 by Dot1 is important for telomeric silencing and Sir protein association. *Genes Dev* 16 1518–1527. 10.1101/gad.1001502 12080090 PMC186335

[B130] Nicholatos J. W. Francisco A. B. Bender C. A. Yeh T. Lugay F. J. Salazar J. E. (2018). Nicotine promotes neuron survival and partially protects from Parkinson’s disease by suppressing SIRT6. *Acta Neuropathol. Commun.* 6:120. 10.1186/s40478-018-0625-y 30409187 PMC6223043

[B131] Nicolas-Avila J. A. Lechuga-Vieco A. V. Esteban-Martinez L. Sanchez-Diaz M. Diaz-Garcia E. Santiago D. J. (2020). A network of macrophages supports mitochondrial homeostasis in the heart. *Cell* 183 94–109.e23. 10.1016/j.cell.2020.08.031 32937105

[B132] Niedernhofer L. J. Gurkar A. U. Wang Y. Vijg J. Hoeijmakers J. H. J. Robbins P. D. (2018). Nuclear genomic instability and aging. *Annu. Rev. Biochem.* 87 295–322. 10.1146/annurev-biochem-062917-012239 29925262

[B133] Nowak R. Siwicki J. K. Chechlinska M. Markowicz S. (2002). Telomere shortening and atherosclerosis. *Lancet* 359:976; author reply 976–977. 10.1016/S0140-6736(02)07997-7 11918940

[B134] Ou H. Schumacher B. (2018). DNA damage responses and p53 in the aging process. *Blood* 131 488–495. 10.1182/blood-2017-07-746396 29141944 PMC6839964

[B135] Palm W. de Lange T. (2008). How shelterin protects mammalian telomeres. *Annu. Rev. Genet.* 42 301–334. 10.1146/annurev.genet.41.110306.130350 18680434

[B136] Pan H. Guan D. Liu X. Li J. Wang L. Wu J. (2016). SIRT6 safeguards human mesenchymal stem cells from oxidative stress by coactivating NRF2. *Cell Res.* 26 190–205. 10.1038/cr.2016.4 26768768 PMC4746611

[B137] Papamichos-Chronakis M. Peterson C. L. (2013). Chromatin and the genome integrity network. *Nat. Rev. Genet.* 14 62–75. 10.1038/nrg3345 23247436 PMC3731064

[B138] Peng L. Qian M. Liu Z. Tang X. Sun J. Jiang Y. (2020). Deacetylase-independent function of SIRT6 couples GATA4 transcription factor and epigenetic activation against cardiomyocyte apoptosis. *Nucleic Acids Res.* 48 4992–5005. 10.1093/nar/gkaa214 32239217 PMC7229816

[B139] Pober J. S. Sessa W. C. (2007). Evolving functions of endothelial cells in inflammation. *Nat. Rev. Immunol.* 7 803–815. 10.1038/nri2171 17893694

[B140] Ravi V. Jain A. Khan D. Ahamed F. Mishra S. Giri M. (2019). SIRT6 transcriptionally regulates global protein synthesis through transcription factor Sp1 independent of its deacetylase activity. *Nucleic Acids Res.* 47 9115–9131. 10.1093/nar/gkz648 31372634 PMC6755095

[B141] Reisländer T. Groelly F. J. Tarsounas M. (2020). DNA damage and cancer immunotherapy: a STING in the tale. *Mol. Cell* 80 21–28. 10.1016/j.molcel.2020.07.026 32810436

[B142] Rezazadeh S. Yang D. Tombline G. Simon M. Regan S. P. Seluanov A. (2019). SIRT6 promotes transcription of a subset of NRF2 targets by mono-ADP-ribosylating BAF170. *Nucleic Acids Res.* 47 7914–7928. 10.1093/nar/gkz528 31216030 PMC6736037

[B143] Rizzo A. Iachettini S. Salvati E. Zizza P. Maresca C. D’Angelo C. (2017). SIRT6 interacts with TRF2 and promotes its degradation in response to DNA damage. *Nucleic Acids Res.* 45 1820–1834. 10.1093/nar/gkw1202 27923994 PMC5389694

[B144] Robin J. D. Ludlow A. T. Batten K. Magdinier F. Stadler G. Wagner K. R. (2014). Telomere position effect: regulation of gene expression with progressive telomere shortening over long distances. *Gene. Dev.* 28 2464–2476. 10.1101/gad.251041.114 25403178 PMC4233240

[B145] Rodier F. Coppé J. Patil C. K. Hoeijmakers W. A. M. Muñoz D. P. Raza S. R. (2009). Persistent DNA damage signalling triggers senescence-associated inflammatory cytokine secretion. *Nat. Cell Biol.* 11 973–979. 10.1038/ncb1909 19597488 PMC2743561

[B146] Rogina B. Helfand S. L. (2004). Sir2 mediates longevity in the fly through a pathway related to calorie restriction. *Proc. Natl. Acad. Sci. U.S.A.* 101 15998–16003. 10.1073/pnas.0404184101 15520384 PMC528752

[B147] Rohini A. Agrawal N. Koyani C. N. Singh R. (2010). Molecular targets and regulators of cardiac hypertrophy. *Pharmacol. Res.* 61 269–280. 10.1016/j.phrs.2009.11.012 19969085

[B148] Rubinsztein D. C. Marino G. Kroemer G. (2011). Autophagy and aging. *Cell* 146 682–695. 10.1016/j.cell.2011.07.030 21884931

[B149] Samani N. J. van der Harst P. (2008). Biological ageing and cardiovascular disease. *Heart* 94 537–539. 10.1136/hrt.2007.136010 18411343

[B150] Saxton R. A. Sabatini D. M. (2017). mTOR signaling in growth, metabolism, and disease. *Cell* 169 361–371. 10.1016/j.cell.2017.03.035 28388417

[B151] Sebastian C. Zwaans B. M. Silberman D. M. Gymrek M. Goren A. Zhong L. (2012). The histone deacetylase SIRT6 is a tumor suppressor that controls cancer metabolism. *Cell* 151 1185–1199. 10.1016/j.cell.2012.10.047 23217706 PMC3526953

[B152] Servillo L. D’Onofrio N. Balestrieri M. L. (2017). Ergothioneine antioxidant function. *J. Cardiovasc. Pharm.* 69 183–191. 10.1097/FJC.0000000000000464 28375902

[B153] Shao J. Yang X. Liu T. Zhang T. Xie Q. R. Xia W. (2016). Autophagy induction by SIRT6 is involved in oxidative stress-induced neuronal damage. *Protein Cell* 7 281–290. 10.1007/s13238-016-0257-6 26983852 PMC4818841

[B154] Shay J. W. (2016). Role of Telomeres and Telomerase in Aging and Cancer. *Cancer Discov.* 6 584–593. 10.1158/2159-8290.CD-16-0062 27029895 PMC4893918

[B155] Shen P. Feng X. Zhang X. Huang X. Liu S. Lu X. (2016). SIRT6 suppresses phenylephrine-induced cardiomyocyte hypertrophy though inhibiting p300. *J. Pharmacol. Sci.* 132 31–40. 10.1016/j.jphs.2016.03.013 27094368

[B156] Shirakabe A. Ikeda Y. Sciarretta S. Zablocki D. K. Sadoshima J. (2016). Aging and autophagy in the heart. *Circ. Res.* 118 1563–1576. 10.1161/CIRCRESAHA.116.307474 27174950 PMC4869999

[B157] Song Y. Shen H. Schenten D. Shan P. Lee P. J. Goldstein D. R. (2012). Aging enhances the basal production of IL-6 and CCL2 in vascular smooth muscle Cells. *Arterioscler. Thromb. Vasc. Biol.* 32 103–109. 10.1161/ATVBAHA.111.236349 22034510 PMC3241880

[B158] Stary H. C. (2000). Natural history and histological classification of atherosclerotic lesions: an update. *Arterioscler. Thromb. Vasc. Biol.* 20 1177–1178. 10.1161/01.atv.20.5.1177 10807728

[B159] Suh J. H. Shenvi S. V. Dixon B. M. Liu H. Jaiswal A. K. Liu R. M. (2004). Decline in transcriptional activity of Nrf2 causes age-related loss of glutathione synthesis, which is reversible with lipoic acid. *Proc. Natl. Acad. Sci. U.S.A.* 101 3381–3386. 10.1073/pnas.0400282101 14985508 PMC373470

[B160] Sundaresan N. R. Vasudevan P. Zhong L. Kim G. Samant S. Parekh V. (2012). The sirtuin SIRT6 blocks IGF-Akt signaling and development of cardiac hypertrophy by targeting c-Jun. *Nat. Med.* 18 1643–1650. 10.1038/nm.2961 23086477 PMC4401084

[B161] Tang S. S. Xu S. Cheng J. Cai M. Y. Chen L. Liang L. L. (2016). Two tagSNPs rs352493 and rs3760908 within SIRT6 gene are associated with the severity of coronary artery disease in a Chinese Han Population. *Dis. Markers* 2016:1628041. 10.1155/2016/1628041 27118880 PMC4826929

[B162] Tang Y. L. Zhou Y. Wang Y. P. Wang J. W. Ding J. C. (2015). SIRT6/NF-kappaB signaling axis in ginsenoside Rg1-delayed hematopoietic stem/progenitor cell senescence. *Int. J. Clin. Exp. Pathol.* 8 5591–5596. 26191269 PMC4503140

[B163] Tao R. Xiong X. DePinho R. A. Deng C. Dong X. C. (2013a). FoxO3 transcription factor and Sirt6 deacetylase regulate low density lipoprotein (LDL)-cholesterol homeostasis via control of the Proprotein Convertase Subtilisin/Kexin Type 9 (Pcsk9) gene expression. *J. Biol. Chem.* 288 29252–29259. 10.1074/jbc.M113.481473 23974119 PMC3795227

[B164] Tao R. Xiong X. DePinho R. A. Deng C. Dong X. C. (2013b). Hepatic SREBP-2 and cholesterol biosynthesis are regulated by FoxO3 and Sirt6. *J. Lipid Res.* 54 2745–2753. 10.1194/jlr.M039339 23881913 PMC3770087

[B165] Tasselli L. Xi Y. Zheng W. Tennen R. I. Odrowaz Z. Simeoni F. (2016). SIRT6 deacetylates H3K18ac at pericentric chromatin to prevent mitotic errors and cellular senescence. *Nat. Struct. Mol. Biol.* 23 434–440. 10.1038/nsmb.3202 27043296 PMC5826646

[B166] Tasselli L. Zheng W. Chua K. F. (2017). SIRT6: novel mechanisms and links to aging and disease. *Trends Endocrinol. Metab.* 28 168–185. 10.1016/j.tem.2016.10.002 27836583 PMC5326594

[B167] Tennen R. I. Bua D. J. Wright W. E. Chua K. F. (2011). SIRT6 is required for maintenance of telomere position effect in human cells. *Nat. Commun.* 2:433. 10.1038/ncomms1443 21847107 PMC3528101

[B168] Tennen R. I. Chua K. F. (2011). Chromatin regulation and genome maintenance by mammalian SIRT6. *Trends Biochem. Sci.* 36 39–46. 10.1016/j.tibs.2010.07.009 20729089 PMC2991557

[B169] Tian K. Liu Z. Wang J. Xu S. You T. Liu P. (2015). Sirtuin-6 inhibits cardiac fibroblasts differentiation into myofibroblasts via inactivation of nuclear factor κB signaling. *Transl. Res.* 165 374–386. 10.1016/j.trsl.2014.08.008 25475987

[B170] Tissenbaum H. A. Guarente L. (2001). Increased dosage of a sir-2 gene extends lifespan in Caenorhabditis elegans. *Nature* 410 227–230. 10.1038/35065638 11242085

[B171] Toiber D. Erdel F. Bouazoune K. Silberman D. M. Zhong L. Mulligan P. (2013). SIRT6 recruits SNF2H to DNA break sites, preventing genomic instability through chromatin remodeling. *Mol. Cell* 51 454–468. 10.1016/j.molcel.2013.06.018 23911928 PMC3761390

[B172] Ungvari Z. Bailey-Downs L. Gautam T. Jimenez R. Losonczy G. Zhang C. (2011a). Adaptive induction of NF-E2-related factor-2-driven antioxidant genes in endothelial cells in response to hyperglycemia. *Am. J. Physiol. Heart C* 300 H1133–H1140. 10.1152/ajpheart.00402.2010 21217061 PMC3075025

[B173] Ungvari Z. Bailey-Downs L. Gautam T. Sosnowska D. Wang M. Monticone R. E. (2011b). Age-associated vascular oxidative stress, Nrf2 dysfunction, and NF-{kappa}B activation in the nonhuman primate *Macaca mulatta*. *J. Gerontol. A Biol. Sci. Med. Sci.* 66 866–875. 10.1093/gerona/glr092 21622983 PMC3148762

[B174] Ungvari Z. Bailey-Downs L. Sosnowska D. Gautam T. Koncz P. Losonczy G. (2011c). Vascular oxidative stress in aging: a homeostatic failure due to dysregulation of NRF2-mediated antioxidant response. *Am. J. Physiol. Heart Circ. Physiol.* 301 H363–H372. 10.1152/ajpheart.01134.2010 21602469 PMC3154665

[B175] Ungvari Z. Orosz Z. Labinskyy N. Rivera A. Xiangmin Z. Smith K. (2007). Increased mitochondrial H2O2 production promotes endothelial NF-kappaB activation in aged rat arteries. *Am. J. Physiol. Heart Circ. Physiol.* 293 H37–H47. 10.1152/ajpheart.01346.2006 17416599

[B176] United Nations. (2019). *World Population Ageing 2019: Highlights (ST/ESA/SER.A/430).* New York, NY: United Nations.

[B177] Valcarcel-Ares M. N. Gautam T. Warrington J. P. Bailey-Downs L. Sosnowska D. de Cabo R. (2012). Disruption of Nrf2 signaling impairs angiogenic capacity of endothelial cells: implications for microvascular aging. *J. Gerontol. A Biol. Sci. Med. Sci.* 67 821–829. 10.1093/gerona/glr229 22219515 PMC3403863

[B178] van der Pol A. van Gilst W. H. Voors A. A. van der Meer P. (2019). Treating oxidative stress in heart failure: past, present and future. *Eur. J. Heart Fail.* 21 425–435. 10.1002/ejhf.1320 30338885 PMC6607515

[B179] Van Gool F. Galli M. Gueydan C. Kruys V. Prevot P. P. Bedalov A. (2009). Intracellular NAD levels regulate tumor necrosis factor protein synthesis in a sirtuin-dependent manner. *Nat. Med.* 15 206–210. 10.1038/nm.1906 19151729 PMC2845476

[B180] Van Meter M. Simon M. Tombline G. May A. Morello T. D. Hubbard B. P. (2016). JNK Phosphorylates SIRT6 to Stimulate DNA double-strand break repair in response to oxidative stress by recruiting PARP1 to DNA breaks. *Cell Rep.* 16 2641–2650. 10.1016/j.celrep.2016.08.006 27568560 PMC5089070

[B181] van Steensel B. Smogorzewska A. de Lange T. (1998). TRF2 protects human telomeres from end-to-end fusions. *Cell* 92 401–413. 10.1016/s0092-8674(00)80932-0 9476899

[B182] Virani S. S. Alonso A. Benjamin E. J. Bittencourt M. S. Callaway C. W. Carson A. P. (2020). Heart disease and stroke statistics-2020 update: a report from the American heart association. *Circulation* 141 e139–e596. 10.1161/CIR.0000000000000757 31992061

[B183] Virmani R. Kolodgie F. D. Burke A. P. Farb A. Schwartz S. M. (2000). Lessons from sudden coronary death: a comprehensive morphological classification scheme for atherosclerotic lesions. *Arterioscler. Thromb. Vasc. Biol.* 20 1262–1275. 10.1161/01.atv.20.5.1262 10807742

[B184] Vitiello M. Zullo A. Servillo L. Mancini F. P. Borriello A. Giovane A. (2017). Multiple pathways of SIRT6 at the crossroads in the control of longevity, cancer, and cardiovascular diseases. *Ageing Res. Rev.* 35 301–311. 10.1016/j.arr.2016.10.008 27829173

[B185] von Zglinicki T. Saretzki G. Docke W. Lotze C. (1995). Mild hyperoxia shortens telomeres and inhibits proliferation of fibroblasts: a model for senescence? *Exp. Cell Res.* 220 186–193. 10.1006/excr.1995.1305 7664835

[B186] Wang J. Uryga A. K. Reinhold J. Figg N. Baker L. Finigan A. (2015). Vascular smooth muscle cell senescence promotes atherosclerosis and features of plaque vulnerability. *Circulation* 132 1909–1919. 10.1161/CIRCULATIONAHA.115.016457 26416809

[B187] Wang L. Ma L. Pang S. Huang J. Yan B. (2016). Sequence variants of SIRT6 gene promoter in myocardial infarction. *Genet. Test Mol. Biomark.* 20 185–190. 10.1089/gtmb.2015.0188 26886147

[B188] Wang T. Sun C. Hu L. Gao E. Li C. Wang H. (2020). Sirt6 stabilizes atherosclerosis plaques by promoting macrophage autophagy and reducing contact with endothelial cells. *Biochem. Cell Biol.* 98 120–129. 10.1139/bcb-2019-0057 31063699

[B189] Wang X. Ria M. Kelmenson P. M. Eriksson P. Higgins D. C. Samnegård A. (2005). Positional identification of TNFSF4, encoding OX40 ligand, as a gene that influences atherosclerosis susceptibility. *Nat. Genet.* 37 365–372. 10.1038/ng1524 15750594

[B190] Wikby A. Nilsson B. O. Forsey R. Thompson J. Strindhall J. Lofgren S. (2006). The immune risk phenotype is associated with IL-6 in the terminal decline stage: findings from the Swedish NONA immune longitudinal study of very late life functioning. *Mech. Ageing Dev.* 127 695–704. 10.1016/j.mad.2006.04.003 16750842

[B191] Winnik S. Auwerx J. Sinclair D. A. Matter C. M. (2015). Protective effects of sirtuins in cardiovascular diseases: from bench to bedside. *Eur. Heart J.* 36 3404–3412. 10.1093/eurheartj/ehv290 26112889 PMC4685177

[B192] Xiao C. Wang R. Lahusen T. J. Park O. Bertola A. Maruyama T. (2012). Progression of chronic liver inflammation and fibrosis driven by activation of c-JUN signaling in Sirt6 mutant mice. *J. Biol. Chem.* 287 41903–41913. 10.1074/jbc.M112.415182 23076146 PMC3516737

[B193] Xiong X. Wang G. Tao R. Wu P. Kono T. Li K. (2016). Sirtuin 6 regulates glucose-stimulated insulin secretion in mouse pancreatic beta cells. *Diabetologia* 59 151–160. 10.1007/s00125-015-3778-2 26471901 PMC4792692

[B194] Xu S. Yin M. Koroleva M. Mastrangelo M. Zhang W. Bai P. (2017). SIRT6 protects against endothelial dysfunction and atherosclerosis in mice. *Aging* 8 1064–1078. 10.18632/aging.100975 27249230 PMC4931854

[B195] Xu Z. Zhang L. Zhang W. Meng D. Zhang H. Jiang Y. (2015). SIRT6 rescues the age related decline in base excision repair in a PARP1-dependent manner. *Cell Cycle* 14 269–276. 10.4161/15384101.2014.980641 25607651 PMC4614943

[B196] Xue M. Joo Y. A. Li S. Niu C. Chen G. Yi X. (2019). Metallothionein protects the heart against myocardial infarction via the mTORC2/FoxO3a/Bim pathway. *Antioxid. Redox Signal.* 31 403–419. 10.1089/ars.2018.7597 30860395

[B197] Yang B. Zwaans B. M. M. Eckersdorff M. Lombard D. B. (2014). The sirtuin SIRT6 deacetylates H3 K56Ac *in vivo* to promote genomic stability. *Cell Cycle* 8 2662–2663. 10.4161/cc.8.16.9329 19597350 PMC2728171

[B198] Yang S. R. Wright J. Bauter M. Seweryniak K. Kode A. Rahman I. (2007). Sirtuin regulates cigarette smoke-induced proinflammatory mediator release via RelA/p65 NF-kappaB in macrophages *in vitro* and in rat lungs *in vivo*: implications for chronic inflammation and aging. *Am. J. Physiol. Lung. Cell Mol. Physiol.* 292 L567–L576. 10.1152/ajplung.00308.2006 17041012

[B199] Ye J. Lenain C. Bauwens S. Rizzo A. Saint-Léger A. Poulet A. (2010). TRF2 and apollo cooperate with topoisomerase 2α to protect human telomeres from replicative damage. *Cell* 142 230–242. 10.1016/j.cell.2010.05.032 20655466

[B200] Yoshizaki T. Milne J. C. Imamura T. Schenk S. Sonoda N. Babendure J. L. (2009). SIRT1 exerts anti-inflammatory effects and improves insulin sensitivity in adipocytes. *Mol. Cell. Biol.* 29 1363–1374. 10.1128/MCB.00705-08 19103747 PMC2643824

[B201] Yu L. M. Dong X. Xue X. D. Xu S. Zhang X. Xu Y. L. (2021). Melatonin attenuates diabetic cardiomyopathy and reduces myocardial vulnerability to ischemia-reperfusion injury by improving mitochondrial quality control: Role of SIRT6. *J. Pineal Res.* 70:e12698. 10.1111/jpi.12698 33016468

[B202] Yu S. Cai Y. Ye J. Pi R. Chen S. Liu P. (2013). Sirtuin 6 protects cardiomyocytes from hypertrophy *in vitro* via inhibition of NF-κB-dependent transcriptional activity. *Br. J. Pharmacol.* 168 117–128. 10.1111/j.1476-5381.2012.01903.x 22335191 PMC3570008

[B203] Yuan R. Tsaih S. W. Petkova S. B. Marin D. E. C. Xing S. Marion M. A. (2009). Aging in inbred strains of mice: study design and interim report on median lifespans and circulating IGF1 levels. *Aging Cell* 8 277–287. 10.1111/j.1474-9726.2009.00478.x 19627267 PMC2768517

[B204] Zaglia T. Milan G. Ruhs A. Franzoso M. Bertaggia E. Pianca N. (2014). Atrogin-1 deficiency promotes cardiomyopathy and premature death via impaired autophagy. *J. Clin. Invest.* 124 2410–2424. 10.1172/JCI66339 24789905 PMC4038560

[B205] Zhang N. Li Z. Mu W. Li L. Liang Y. Lu M. (2016). Calorie restriction-induced SIRT6 activation delays aging by suppressing NF-κB signaling. *Cell Cycle* 15 1009–1018. 10.1080/15384101.2016.1152427 26940461 PMC4889297

[B206] Zhang Q. Tu W. Tian K. Han L. Wang Q. Chen P. (2019). Sirtuin 6 inhibits myofibroblast differentiation via inactivating transforming growth factor-beta1/Smad2 and nuclear factor-kappaB signaling pathways in human fetal lung fibroblasts. *J. Cell. Biochem.* 120 93–104. 10.1002/jcb.27128 30230565

[B207] Zhang W. Qu X. Chen B. Snyder M. Wang M. Li B. (2016). Critical roles of STAT3 in β-adrenergic functions in the heart. *Circulation* 133 48–61. 10.1161/CIRCULATIONAHA.115.017472 26628621 PMC4698100

[B208] Zhang W. Wan H. Feng G. Qu J. Wang J. Jing Y. (2018). SIRT6 deficiency results in developmental retardation in cynomolgus monkeys. *Nature* 560 661–665. 10.1038/s41586-018-0437-z 30135584

[B209] Zhang W. Wei R. Zhang L. Tan Y. Qian C. (2017). Sirtuin 6 protects the brain from cerebral ischemia/reperfusion injury through NRF2 activation. *Neuroscience* 366 95–104. 10.1016/j.neuroscience.2017.09.035 28951325

[B210] Zhang X. Li W. Shen P. Feng X. Yue Z. Lu J. (2016). STAT3 suppression is involved in the protective effect of SIRT6 against Cardiomyocyte hypertrophy. *J. Cardiovasc. Pharm.* 68 204–214. 10.1097/FJC.0000000000000404 27124607

[B211] Zhang Y. Sowers J. R. Ren J. (2018). Targeting autophagy in obesity: from pathophysiology to management. *Nat. Rev. Endocrinol.* 14 356–376. 10.1038/s41574-018-0009-1 29686432

[B212] Zhang Z. Ren S. Tan Y. Li Z. Tang X. Wang T. (2016). Epigenetic regulation of NKG2D ligands is involved in exacerbated atherosclerosis development in Sirt6 heterozygous mice. *Sci. Rep.* 6:23912. 10.1038/srep23912 27045575 PMC4820703

[B213] Zhang Z. Z. Cheng Y. W. Jin H. Y. Chang Q. Shang Q. H. Xu Y. L. (2017). The sirtuin 6 prevents angiotensin II-mediated myocardial fibrosis and injury by targeting AMPK-ACE2 signaling. *Oncotarget* 8 72302–72314. 10.18632/oncotarget.20305 29069788 PMC5641131

[B214] Zhao Q. D. Viswanadhapalli S. Williams P. Shi Q. Tan C. Yi X. (2015). NADPH oxidase 4 induces cardiac fibrosis and hypertrophy through activating Akt/mTOR and NFkappaB signaling pathways. *Circulation* 131 643–655. 10.1161/CIRCULATIONAHA.114.011079 25589557 PMC4568756

[B215] Zhong L. D’Urso A. Toiber D. Sebastian C. Henry R. E. Vadysirisack D. D. (2010). The histone deacetylase Sirt6 regulates glucose homeostasis via Hif1alpha. *Cell* 140 280–293. 10.1016/j.cell.2009.12.041 20141841 PMC2821045

